# Molecular mechanisms of coronary disease revealed using quantitative trait loci for TCF21 binding, chromatin accessibility, and chromosomal looping

**DOI:** 10.1186/s13059-020-02049-5

**Published:** 2020-06-08

**Authors:** Quanyi Zhao, Michael Dacre, Trieu Nguyen, Milos Pjanic, Boxiang Liu, Dharini Iyer, Paul Cheng, Robert Wirka, Juyong Brian Kim, Hunter B. Fraser, Thomas Quertermous

**Affiliations:** 1 Division of Cardiovascular Medicine and Cardiovascular Institute, Stanford University School of Medicine, 300 Pasteur Dr. Falk CVRC, Stanford, CA 94305 USA; 2 Department of Biology, Stanford University, Stanford, CA 94305 USA

**Keywords:** Coronary artery disease, Smooth muscle cells, Quantitative trait locus, TCF21, Chromosomal looping, Chromatin accessibility

## Abstract

**Background:**

To investigate the epigenetic and transcriptional mechanisms of coronary artery disease (CAD) risk, as well as the functional regulation of chromatin structure and function, we create a catalog of genetic variants associated with three stages of transcriptional *cis*-regulation in primary human coronary artery vascular smooth muscle cells (HCASMCs).

**Results:**

We use a pooling approach with HCASMC lines to map regulatory variants that mediate binding of the CAD-associated transcription factor TCF21 with ChIPseq studies (bQTLs), variants that regulate chromatin accessibility with ATACseq studies (caQTLs), and chromosomal looping with Hi-C methods (clQTLs). We examine the overlap of these QTLs and their relationship to smooth muscle-specific genes and transcription factors. Further, we use multiple analyses to show that these QTLs are highly associated with CAD GWAS loci and correlate to lead SNPs where they show allelic effects. By utilizing genome editing, we verify that identified functional variants can regulate both chromatin accessibility and chromosomal looping, providing new insights into functional mechanisms regulating chromatin state and chromosomal structure. Finally, we directly link the disease-associated TGFB1-SMAD3 pathway to the CAD-associated FN1 gene through a response QTL that modulates both chromatin accessibility and chromosomal looping.

**Conclusions:**

Together, these studies represent the most thorough mapping of multiple QTL types in a highly disease-relevant primary cultured cell type and provide novel insights into their functional overlap and mechanisms that underlie these genomic features and their relationship to disease risk.

## Background

While there has been considerable success identifying loci in the human genome that are associated with a broad range of human diseases, including coronary artery disease (CAD) [1–3], most identified regions represent non-exonic regulatory sequence and are thus difficult to associate to a particular gene or functional annotation of the genome. Toward this end, numerous studies have explored the genetics of gene expression and mapped expression quantitative trait locus variants to inform on which SNPs and which genes are causally related to disease in the associated loci [4–7]. A number of these expression quantitative trait variants (eQTLs) have been found to be the causal variants in disease loci and in general promoted causal gene discovery [8].

The regulatory variants that modulate other genomic features have also been characterized. QTLs for histone modification and chromatin accessibility and more recently chromosomal looping have also been mapped [9–12]. Such efforts have provided important information regarding the regulatory structure of the non-coding sequence in the genome and identified how such variation may regulate the risk for complex human disease. In general, these studies have required conducting time-consuming and costly assays for each of the large numbers of individuals that is required to reach relevant genome-wide levels of statistical association. Recently, we have developed an efficient approach for mapping molecular QTLs which employs sample pooling prior to sequencing, and employed this method to map QTLs for transcription factor binding [13], methylation [14], and chromatin accessibility [9]. This pooling minimizes experimental variability between samples (both within and between experimental “batches”), considerably reducing the cost and effort of QTL mapping when compared to standard individual-level approaches [13].

To better understand the functional basis of regulatory features of the human genome and to accelerate understanding of the transcriptional mechanisms by which these features contribute to CAD, we have mapped quantitative trait variation in disease-relevant primary cultured human coronary artery vascular smooth muscle cells (HCASMCs). We have identified QTLs for binding of the CAD-associated transcription factor TCF21 (bQTLs), chromatin accessibility (caQTLs), and chromosomal looping (clQTLs) and investigated their relationship to one another and to eQTLs mapped in the same cell type, as well as their overlap with CAD-associated genetic variation.

## Results

### bQTL, caQTL, and clQTL calling in pooled HCASMC lines

**Fig. 1:**
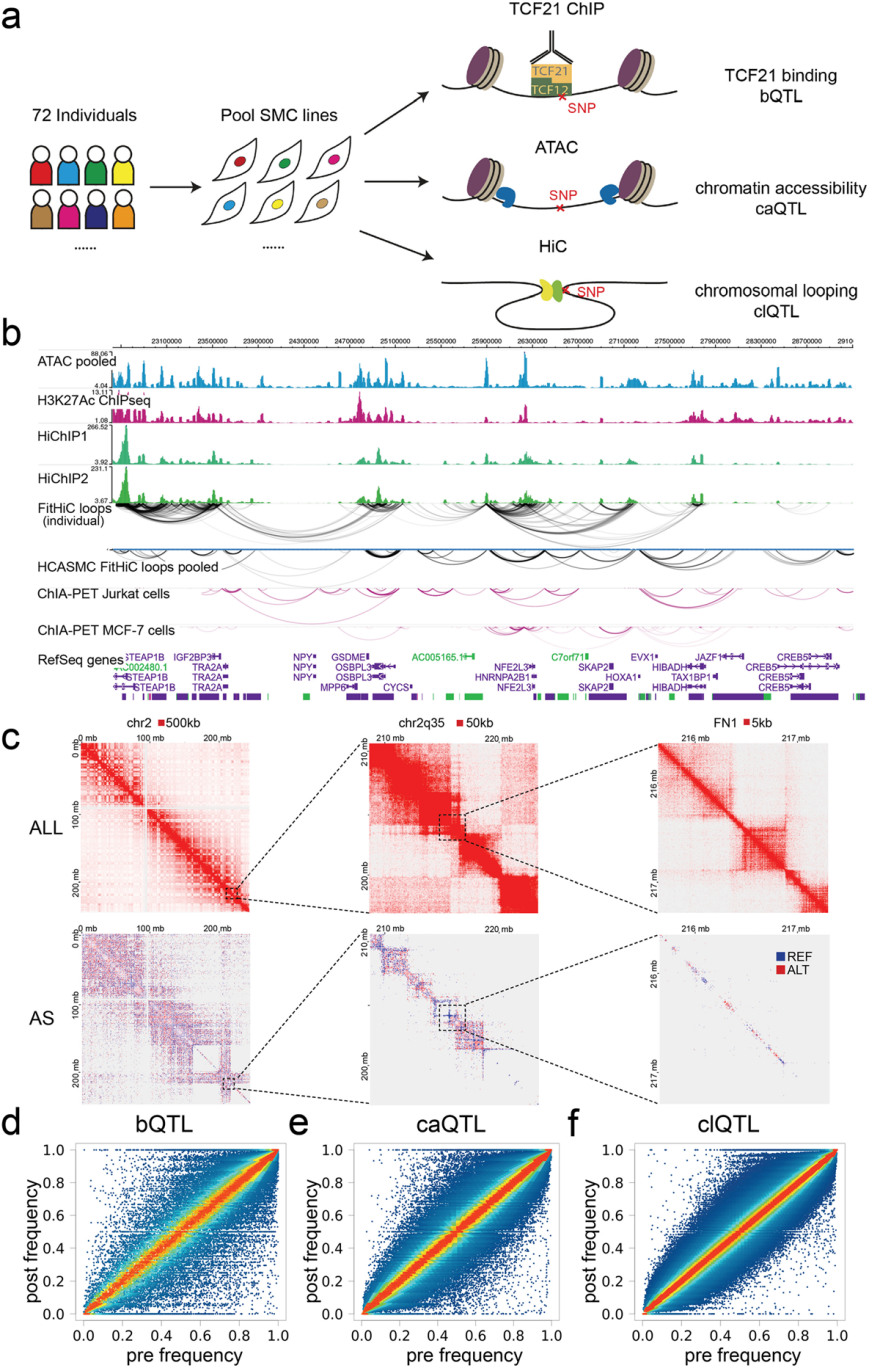
bQTLs, caQTLs, and clQTLs in pooled HCASMC lines. **a** Diagram showing the approaches for pooled TCF21 ChIPseq, ATACseq, Hi-C, and QTL calling. **b** Genome browser session showing the HCASMC pooled sequencing tracks, loops called from Hi-C, and their comparison with HiChIP in an individual line or non-SMC ChIA-PET. **c** Heatmaps show the chromosomal contacts on chr2 at a variety of magnifications and resolutions. ALL, all loops; AS, allele-specific loops. **d** Plots showing the regression results and pre-post frequency distributions for TCF21 binding, 5315 bQTLs, **e** chromatin accessibility, 8346 caQTLs, and **f** chromosomal looping, 7084 clQTLs

We obtained primary HCASMC lines from commercial vendors. Genotypes were called with whole-genome sequencing or genotyping with Illumina chips, phased, and imputed against 1000 Genome phase 3 data before merging [15]. We pooled 65 lines for TCF21 ChIPseq and Hi-C and 71 lines for ATACseq experiments from total 72 lines at passages 4–6 (Fig. 1a, the “Methods” section). After sequencing, we obtained more than 400 M reads each for pooled TCF21 ChIPseq and ATACseq libraries and 800 M reads for the pooled Hi-C library. Standard pipelines were employed for each of these genomic approaches. We called 22,381 high-confidence binding peaks from TCF21 ChIPseq with fold enrichment > 10, *P* value < 10^−20^ cutoffs, and 18,601 open chromatin regions from ATACseq with fold enrichment > 4, *P* value < 10^−10^. Most of these TCF21 binding peaks (19,705, > 88%) and open chromatin regions (17,129, > 92%) overlapped with the data previously identified in an individual HCASMC line [16]. For the Hi-C data, in addition to standard data processing, we also assigned sequencing reads to the SNPs genotyped in our pooled HCAMSC lines, generating a total of more than 377 million (M) valid interacted reads pairs. Of these, approximately 21.6 M allele-specific interactions with at least one SNP inside the loop boundaries were identified (Additional file 1: Suppl Fig. 1a, b). Using these interactions, we were able to find 7916 loops by FitHiC and 3443 loops by HiCCUPS with a stringent *P* value cutoff (10^−10^). Here we show an example genome region at 7p15.2, comparing our pooled ATACseq track and Hi-C loops for heterologous cell types with our previously published H3K27ac HiChIP loops in an individual HCASMC line [17], and ENCODE generated ChIA-PET loops (Fig. 1b). Our pooled Hi-C loops showed similar patterns but with a higher resolution and more chromosomal contacts compared to the previously reported data [17]. More importantly, our data showed high resolution (5 kb) of allele-specific interactions, revealing the differential chromosomal architectures associated with individual SNPs (Fig. 1c).

To call the TCF21 binding (bQTL), chromatin accessibility (caQTL), and chromosomal looping (clQTL) quantitative trait loci, we employed a published regression-based approach which uses post-assay allele frequencies together with genotypes of each sample to infer the proportion of each sample in the pool [9, 13]. Comparing the pre-assay vs. post-assay allele frequencies allows the identification of outlier SNPs where these frequencies are significantly different from one another, which indicates a *cis*-acting QTL variant. With fold enrichment optimized [9] at *P* value cutoff 10^−3^, we obtained 5315 significant bQTLs, 8346 caQTLs, and 7084 clQTLs with the regression analysis from total 60,706, 455,165, and 231,7347 tested variants, respectively (Fig. 1d–f, Additional file 2: Suppl Table 1). To verify the significance of our pooling and QTL mapping results, we intersected our caQTLs with the previously reported caQTLs in lymphoblastoid cell lines (LCLs) [9]. With *P* value cutoff 10^−3^, our caQTLs have 20.4% (1705) overlap with LCL QTLs. There was at most 32.9% (2745) overlap with *P* value cutoff 10^−2^, suggesting that regulatory variation is quite different between these two cell types and underscoring the importance of these smooth muscle cell data for the study of vascular disease genetics.

### QTLs are highly associated with GWAS CAD loci

An initial question we wanted to address was the relationship of mapped QTLs with coronary artery disease (CAD)-associated variation. To examine this question, we used GWAS catalog SNPs [18] supplemented with CARDIoGRAMplusC4D variant data from a 1000 Genomes-based meta-analysis [19]. First, we extracted lead SNPs in CAD-associated categories “Atherosclerosis,” “Coronary artery calcification,” “Coronary artery,” “Coronary heart,” “Kawasaki disease,” and “Myocardial infarction” from the GWAS catalog along with the lead SNPs in CARDIoGRAMplusC4D. We also randomly selected 27 unrelated diseases to serve as background. We then investigated the enrichment of these lead SNPs in a ± 1-kb window around the QTLs.

**Fig. 2:**
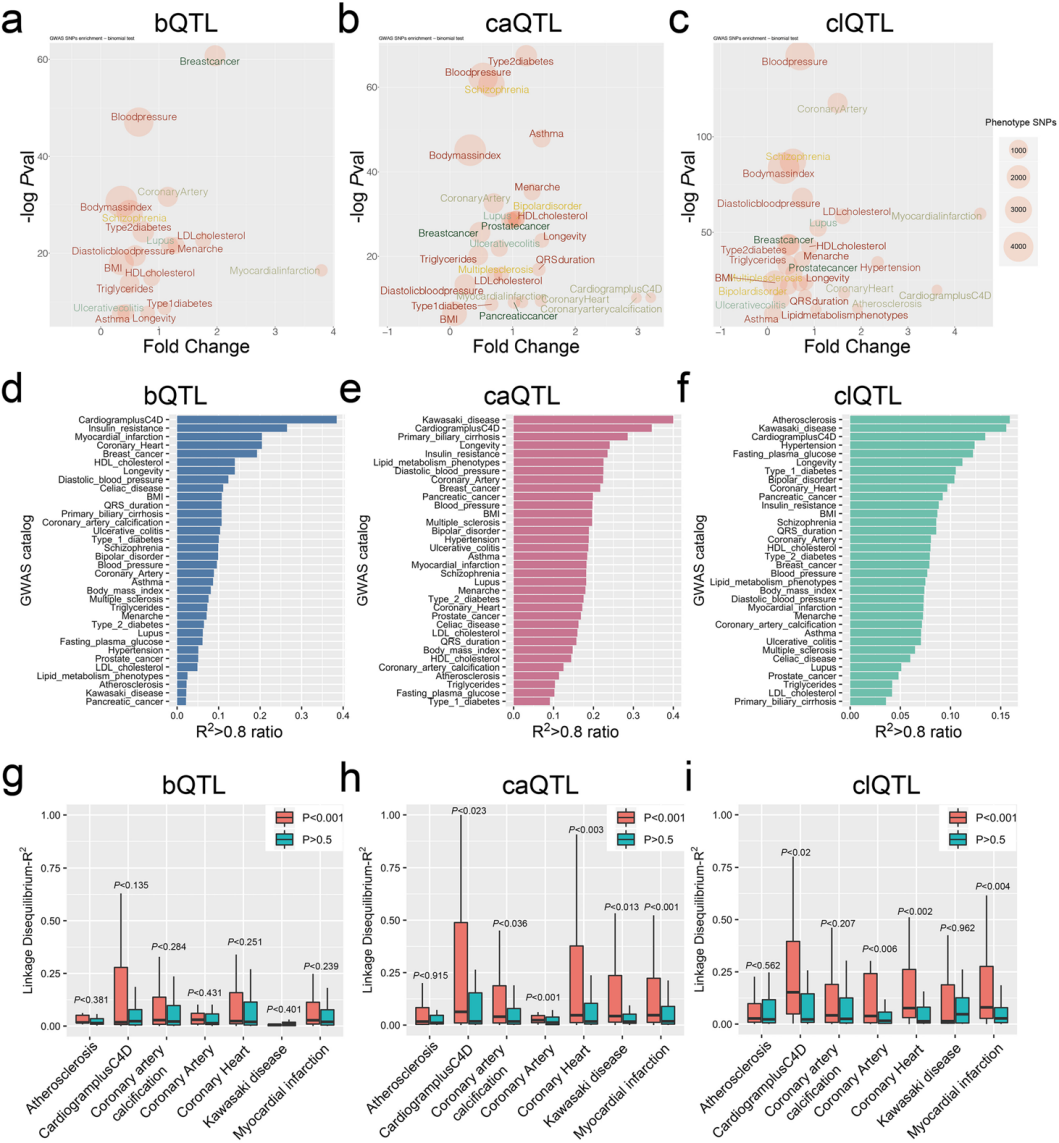
QTLs are highly associated with GWAS CAD loci. **a** bQTL, **b** caQTL, and **c** clQTL plots show the lead SNPs of GWAS catalog enrichment in the ± 1-kb windows of the QTLs, including *P* values, fold changes, and number sizes. The colors of the text indicate the different categories of diseases. **d** bQTL, **e** caQTL, and **f** clQTL bar graphs show the ranked GWAS terms for associated lead SNP-QTL pairs with linkage disequilibrium (LD) (*R*^2^ > 0.8) in the GWAS catalog, compared to the total number of pairs. **g** bQTL, **h** caQTL, and **i** clQTL box plots show the total LD *R*^2^ distributions of QTL to lead SNP pairs, in GWAS CAD-associated diseases

This analysis revealed that the lead SNPs in CAD-associated categories have a strong overlap (*P* < 10^−10^) with the bQTLs, caQTLs, and clQTLs. The highest enriched category for bQTL variants was “Myocardial infarction” and for caQTLs “CARDIoGRAMplusC4D” and “Coronary artery calcification.” clQTL variants were enriched for “Myocardial infarction,” “CardiogramplusC4D,” and the highly significant term “Coronary artery,” which was the second most significant category (*P* < 10^−100^) ranked by *P* value (Fig. 2a–c). This finding suggested that these QTLs in HCASMC may play an important role in the genetic mechanism of HCASMC-mediated disease progression. All of the QTLs showed enrichment for blood pressure and hypertension phenotypes which are highly consistent with known SMC functions, and association of *TCF21* with blood pressure has been identified by population studies with multiple racial ethnic groups [20–22]. The highly significant enrichment of breast cancer variants in the bQTL category is consistent with TCF21 being a known tumor suppressor and that is dysregulated in breast cancer, but the enrichment of breast cancer variants among the caQTLs and clQTLs is surprising and suggests a similar genomic and genetic architecture between HCASMC and breast cancer risk genes [23].

To further validate these results, we also evaluated the association of QTLs with disease risk variants by linkage disequilibrium (LD) comparison. We selected all of the GWAS lead SNP-QTL pairs which were separated by less than 100 kb, calculated the *R*^2^ for each pair, and then ranked the categories by the ratio of pairs with *R*^2^ > 0.8 compared to the total number of pairs. Each of the three types of QTLs showed CAD-related categories as among the three most significant (Fig. 2d–f). Interestingly, these analyses also showed some QTL specificity: “Myocardial infarction” is enriched in bQTL and “Atherosclerosis” only in clQTL, while “Kawasaki disease” is enriched only for caQTL and clQTL variants (Fig. 2d–f). These results were consistent with the distance-dependent enrichment results (Fig. 2a–c).

We next focused on the CAD-associated categories only, comparing the *R*^2^ distributions of the LD pairs to the significant QTLs (*P* < 10^−3^) with those of non-significant SNPs (*P* > 0.5). While the bQTLs showed a trend in the correct direction, they did not show a significant correlation (Fig. 2g). However, the caQTLs (Fig. 2h) and clQTLs (Fig. 2i) showed a statistically greater correlation than non-significant SNPs. The fold change of *R*^2^ > 0.8 pairs of significant to non-significant QTLs indicated enrichments for the majority of the cardiovascular diseases (Additional file 1: Suppl Fig. 2a, b, and c). These data demonstrate that the QTLs we identified are significantly enriched in CAD-associated loci.

### QTLs colocate with SMC transcription factor binding and epigenetic modifications

**Fig. 3:**
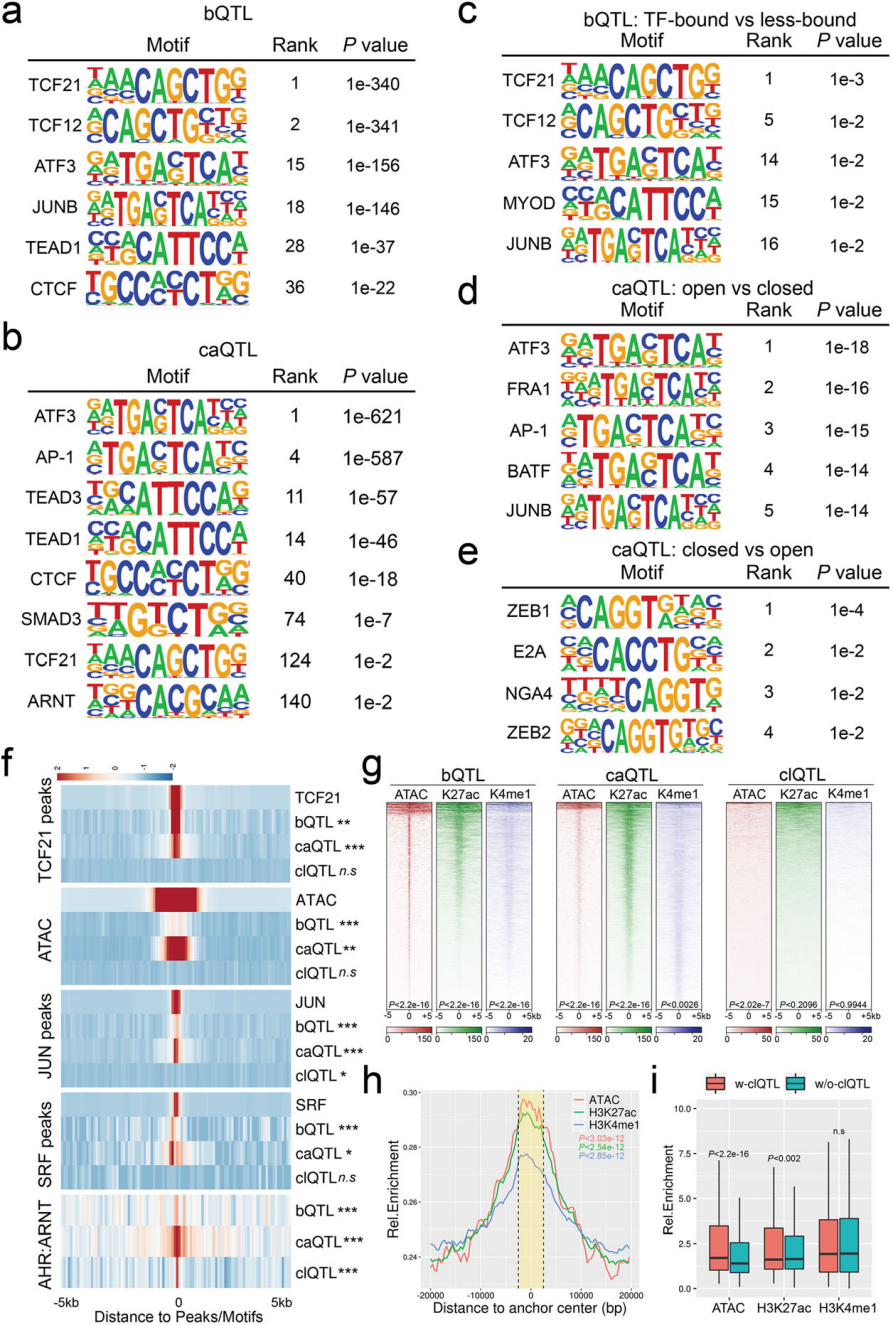
SMC relevant transcription factors are colocalized with QTLs. Results from HOMER reference motif scanning on ± 50-bp windows surrounding **a** bQTLs and **b** caQTLs. Allele-specific motif scanning with flipped SNPs in ± 20-bp windows surrounding **c** bound-vs-less-bound bQTLs, **d** open-vs-closed caQTLs, and **e** closed-vs-open caQTLs. **f** Heatmaps show the bQTL, caQTL, and clQTL densities at TCF21 peaks, ATAC peaks, JUN peaks, SRF peaks, and AHR:ARNT binding motifs. ***P < 10^−8^; **P < 10^−4^; *P < 10^−2^; ^n.s^*P* > 0.01. **g** Heatmaps show the ATACseq open chromatin (ATAC), histone H3K27ac (K27ac), and H3K4me1(K4me1) mark enrichment at bQTLs, caQTLs, and clQTLs in ± 5-kb windows. **h** Density plot showing open chromatin, H3K27ac, and H3K4me1 enrichment at chromosomal loop anchors (yellow area within dashed lines). **i** Boxplots show differences between the enrichments of open chromatin, H3K27ac, and H3K4me1, on chromosomal loop anchors with and without clQTLs

We have previously shown that open chromatin regions in HCASMC are enriched for CAD-associated loci and multiple SMC-specific transcription factor (TF) binding sites, such as TCF21 [24]. We extended these studies by investigating TF motif enrichment analysis at the identified QTLs. We created a window of analysis that extended 50 bp upstream and downstream of each significant (*P* < 10^−3^) QTL and scanned these sequences by matching to the HOMER known motifs or JASPAR core 2018 vertebrate database [25]. Non-significant QTLs (*P* > 0.5) with the same window size served as background. The most enriched motifs around bQTLs were TCF21 and its bHLH Class I dimerization partner TCF12 (Fig. 3a). The AP-1 complex subunit (ATF3 and JUNB), the Hippo pathway transcription factor TEAD1, and the chromatin regulator CTCF motifs were also enriched in these regions of the genome, suggesting that they could be regulated together with TCF21 binding at these loci (Fig. 3a). For caQTLs, we found that the AP-1 complex is the dominant TF motif nearby, along with those for SMC functional TFs such as TEAD1/3, SMAD3, TCF21, and ARNT (Fig. 3b). These enrichments and colocations extend our previous studies [16, 24]. A similar analysis was also performed with clQTLs, which showed ZEB1 and ZEB2 binding site enrichment and also enrichment for a number of TF motifs that have not been reported to be associated with CAD or SMC function (Additional file 1: Suppl Fig. 3a, b).

To investigate the allele-specific TF binding at these QTLs, we searched the motifs that were differentially enriched among the high-chromatin accessibility or TCF21-bound alleles compared to the low-chromatin accessibility or TCF21 less-bound alleles for the same QTLs in a ± 20-bp window. The results show that TCF21/TCF12, AP-1 complex motifs, and a bHLH E-box assigned to MYOD are only enriched in open bQTL alleles with no significant TF motifs found in closed alleles (Fig. 3c). The AP-1 complex motif was associated preferentially with open caQTL alleles, presumably through pioneer functions that promote chromatin accessibility (Fig. 3d) [16]. Interestingly, we found accessible ZEB1 and ZEB2 binding sites at closed caQTL alleles, suggesting that they may have the opposite function of AP-1 in SMC (Fig. 3e) [26].

We generated heatmaps to investigate colocalization of the QTLs with each other, and with HCASMC ATACseq and ChIPseq data. TCF21 bQTLs were shown to colocalize with previous ChIPseq data [27] (Fig. 3f) in regions of open chromatin that mediate binding of the SMC-related MYOCD-SRF complex [28] and JUN [16], supporting the authenticity of the bQTLs. Additionally, we found ATACseq open chromatin, as well as histone H3K27ac and H3K4me1 marks, to be highly enriched at bQTLs (Fig. 3f, g), suggesting that they may regulate TCF21 binding by affecting the epigenome and chromatin accessibility. As expected, caQTLs were enriched at ATACseq regions of open chromatin and further localized to enhancer regions (Fig. 3f, g). With the exception of AHR:ARNT binding motifs, clQTLs in general showed minimal colocation with other QTLs or ChIPseq peaks for TFs or histone modifications (Fig. 3f, g). However, chromosomal loops were enriched at their anchors for chromatin accessibility as well as H3K27ac and H3K4me1 chromatin marks (Fig. 3h), with those loops having clQTLs in their anchors showing higher chromatin accessibility and H3K27ac levels than those loops without clQTLs (Fig. 3i). Taken together, these data show that variation regulating TCF21 binding colocalizes with caQTLs in regions of open chromatin that have enhancer histone marks and are enriched for clQTLs at looping anchors, suggesting that epigenetic effects mediate a significant proportion of genomic molecular phenotypes.

### Overlapping QTLs identify genes that mediate key SMC functional roles

**Fig. 4:**
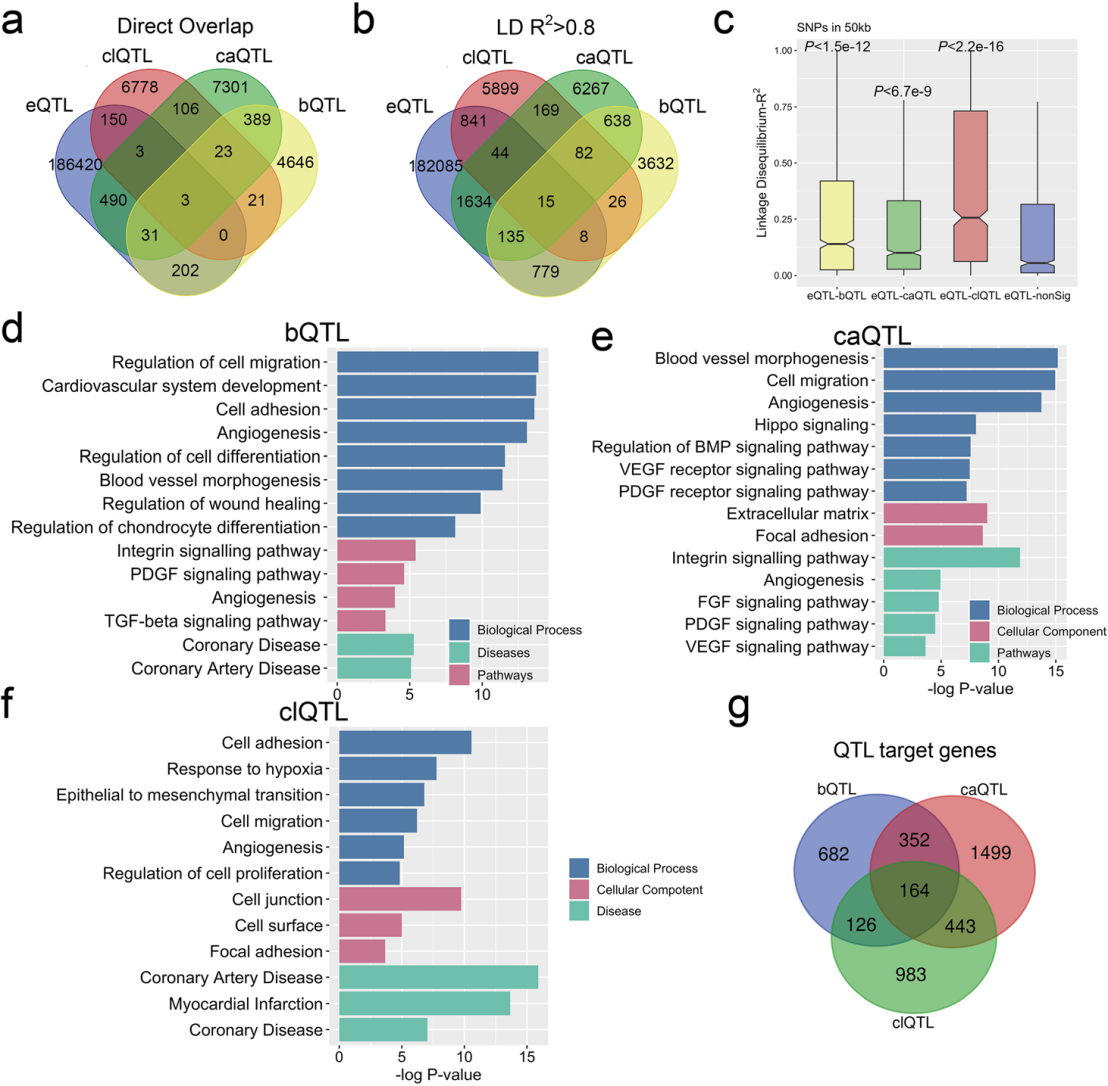
Colocalizing QTLs identify genes that mediate key roles in SMC functions. Venn diagrams show **a** direct overlaps or **b** linkage disequilibrium (LD) *R*^2^ > 0.8 between bQTL, caQTL, clQTL, and eQTLs all mapped in HCASMC. **c** Box plots show LD *R*^2^ distributions between bQTL-eQTL, caQTL-eQTL, and clQTL-eQTL pairs. Bar graphs show Gene Ontology analysis of target genes of **d** bQTLs, **e** caQTLs, and **f** clQTLs within 50 kb. **g** The Venn diagram shows the overlap between bQTL-, caQTL-, and clQTL-associated genes

We hypothesized that QTLs for the different molecular traits would collocate in a number of loci and that these loci would show a greater likelihood of identifying genes and pathways that support specific functions of SMC and their relationship to vascular disease. Our next goal was thus to characterize loci with bQTL, caQTL, and clQTL colocation. We first overlapped these QTLs to examine exact matching and included our published HCASMC eQTL data in this analysis [15]. The number of eQTLs (187299) was comparable to GTEx QTL numbers and QTL-gene pairs (Additional file 1: Suppl Fig. 4a, b). We found 446 direct overlaps between bQTLs and caQTLs, 135 between caQTLs and clQTLs, 47 between bQTLs and clQTLs, and 26 overlaps for all three (Fig. 4a). Each QTL group had numerous exact matches with eQTLs while there were few overlaps for all four QTL groups. Interestingly, the overlaps between bQTLs and caQTLs were found to be directional. TF-bound alleles of bQTLs had more matches with open alleles of caQTLs than those with closed alleles while a comparison of less-bound alleles of bQTLs with caQTLs showed opposite results (Additional file 1: Suppl Fig. 4c). We attribute these findings to the overlap of TCF21 binding and open chromatin regions as we previously described (Fig. 3g, h) [16].

Further, we used LD *R*^2^ > 0.8 instead of exact matching to determine overlap. As expected, the overlaps among bQTLs, caQTLs, and clQTLs and their overlaps with eQTLs all increased (Fig. 4b). To further characterize the associations between eQTLs, bQTLs, caQTLs, and clQTLs, we calculated the *R*^2^ of all the LD pairs within 100 kb maximum distance and found that the *R*^2^ distributions of eQTL to significant (*P* < 10^−3^) bQTLs, caQTLs, or clQTLs were statistically higher than those to non-significant (*P* > 0.5) QTLs (Fig. 4c, Additional file 1: Suppl Fig. 4d, e and f). These data suggest that these QTLs may share a group of the same target genes with eQTLs and could potentially provide a mechanism for their transcriptional regulation.

We next assigned the QTLs to their target genes using our chromatin interaction Hi-C data (the “Methods” section). Gene Ontology analysis of the identified genes showed a strong association of these genes with SMC functions, including “extracellular matrix organization,” “blood vessel morphogenesis,” “focal adhesion,” “angiogenesis,” “cell differentiation,” “cell division,” “coronary artery disease,” and “myocardial infarction” (Fig. 4d–f). The targets of these three QTL groups share 98 genes, which have similar gene ontology results as the individual analyses (Fig. 4g, Additional file 1: Suppl Fig. 4g). These data provide a group of novel candidate loci that may contribute to CAD and were examined further in the following studies.

### QTLs located in CAD GWAS causal loci show allele-specific TCF21 binding, chromatin accessibility, and chromosomal looping

To investigate the functional association of the HCASMC QTLs with expression of genes in CAD loci, we pursued mechanistic studies to characterize their transcriptional relationship. Given the association of the three types of QTLs with GWAS CAD loci and the QTL target genes with SMC functions, we intersected these CAD SNPs with the QTL colocalized loci and then searched their nearest genes. We found 86 QTLs that associate with 151 CAD SNPs. These SNPs are located in 62 loci across the genome (Additional file 1: Suppl Fig. 5a left). After evaluating the corresponding genes according to published studies, we selected 36 candidate causal genes that are highly likely to be associated with CAD risk (Additional file 3: Suppl Table 2). We then separated them into three groups by their distances to CAD SNPs and QTLs (Additional file 1: Suppl Fig. 5a right). Overall, most of these genes were expressed in both GTEx coronary artery tissues (Additional file 1: Suppl Fig. 5b) and HCASMC (Additional file 1: Suppl Fig. 5c). We further investigated the genes in the first group to derive our validation candidates.

**Fig. 5:**
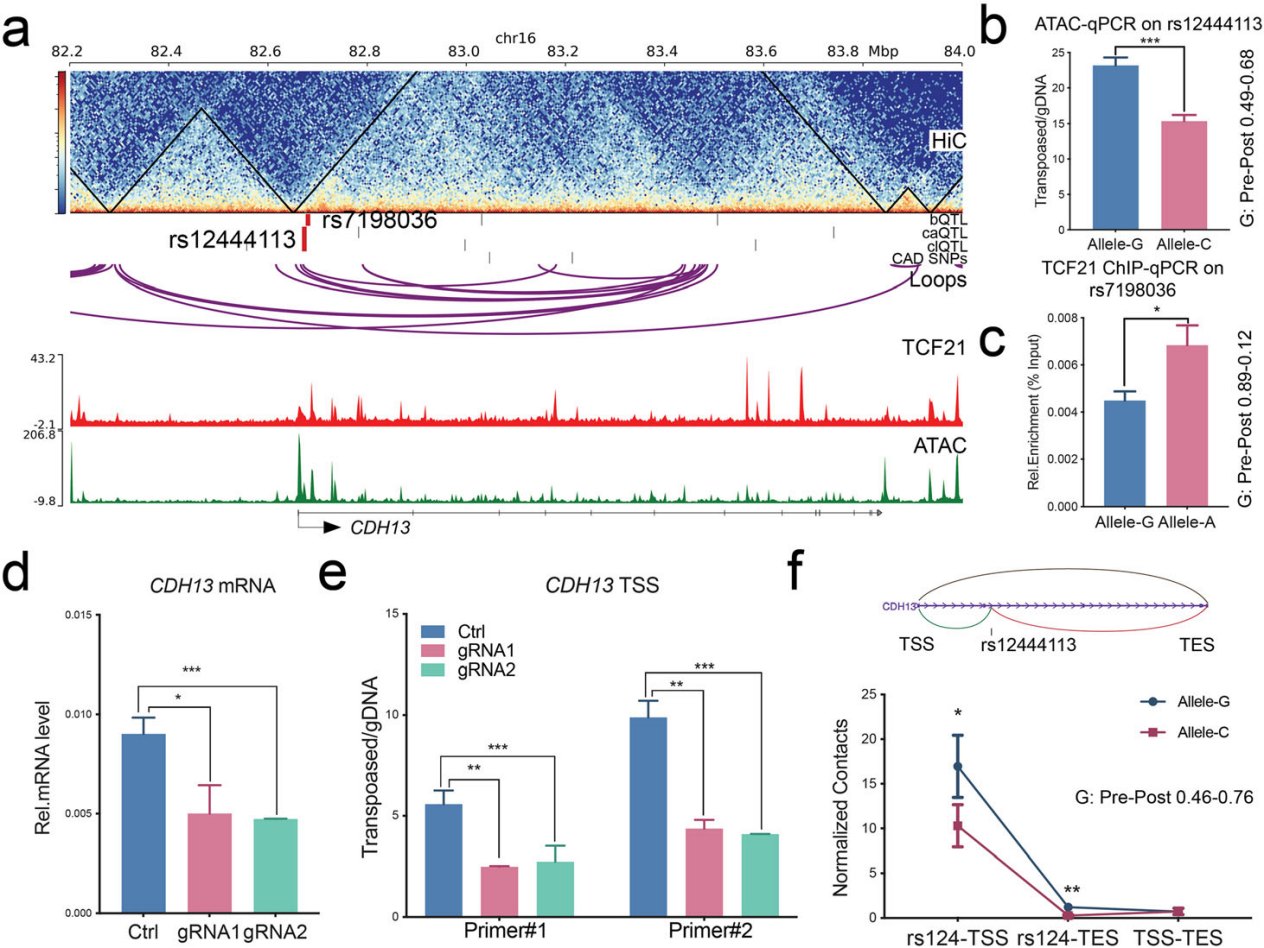
QTLs located in GWAS gene *CDH13* show allele-specific TCF21 binding, chromatin accessibility, and chromosomal looping. **a***CDH13* locus, showing bQTL rs7198036 and ca/clQTL rs12444113. Bar graphs show allelic enrichments of **b** chromatin accessibility at rs12444113 and **c** TCF21 binding at rs7198036 identified by allele-specific qPCR, **d** mRNA levels, and **e** chromatin accessibility at the TSS region of *CDH13* suppressed by CRISPRi-KRAB targeting at rs12444113. **f** Diagram (top) shows the chromosomal loops between rs12444113 and TSS/TES of *CDH13*. 3C-PCR (bottom) shows the differential chromosomal contacts from rs12444113 to TSS and TES of *CDH13*. Pre-post: allele frequencies in the QTL regression data. Shown are means ± SD; *n* = 3; ****P < 0.0001; ***P < 0.001; **P < 0.01; *P < 0.05

These genes, *ARNTL*, *CCBE1*, *CDH13*, *EMP1*, and *FN1*, have been reported as CAD-associated, or related to vascular development or function [2, 19, 29]. In addition, they are involved in epithelial–mesenchymal transition (EMT) processes, which are prominent in vascular disease [30–33]. We validated the QTLs in *CDH13* and *FN1* loci with multiple approaches. For *CDH13*, bQTL rs7198036 and the combination caQTL and clQTL rs12444113 were noted to be located ~ 10 kb downstream of the transcription start site (TSS) (Fig. 5a). We performed ATAC-qPCR and ChIP-qPCR in HCASMC heterozygous lines for these QTLs using Taqman genotyping primers and confirmed that the G allele had higher chromatin accessibility than the C allele at rs12444113 (Fig. 5b) and allele A had a higher TCF21 occupancy than allele G at rs7198036 (Fig. 5c). To verify the clQTL allele specificity, we employed dCas9-KRAB inhibition “CRISPRi” and investigated *CDH13* expression. The two gRNAs targeting rs12444113 efficiently reduced *CDH13* transcription (Fig. 5d) and chromatin accessibility at the *CDH13* TSS region (Fig. 5e). Since the QTLs are located more than 10 kb from the TSS, it is more likely to be a long-range regulatory effect rather than a local *cis*-effect. To further confirm this, we designed an allele-specific 3C-PCR assay to detect the chromosomal interactions with different alleles using HCASMC heterozygous lines. The data confirms that rs12444113 has much greater contact with the *CDH13* TSS than the transcription end site (TES), while they both show allele specificity (Fig. 5f). The measured imbalance for these alleles was consistent with the pre-post allele frequencies in the QTL regression data.

**Fig. 6:**
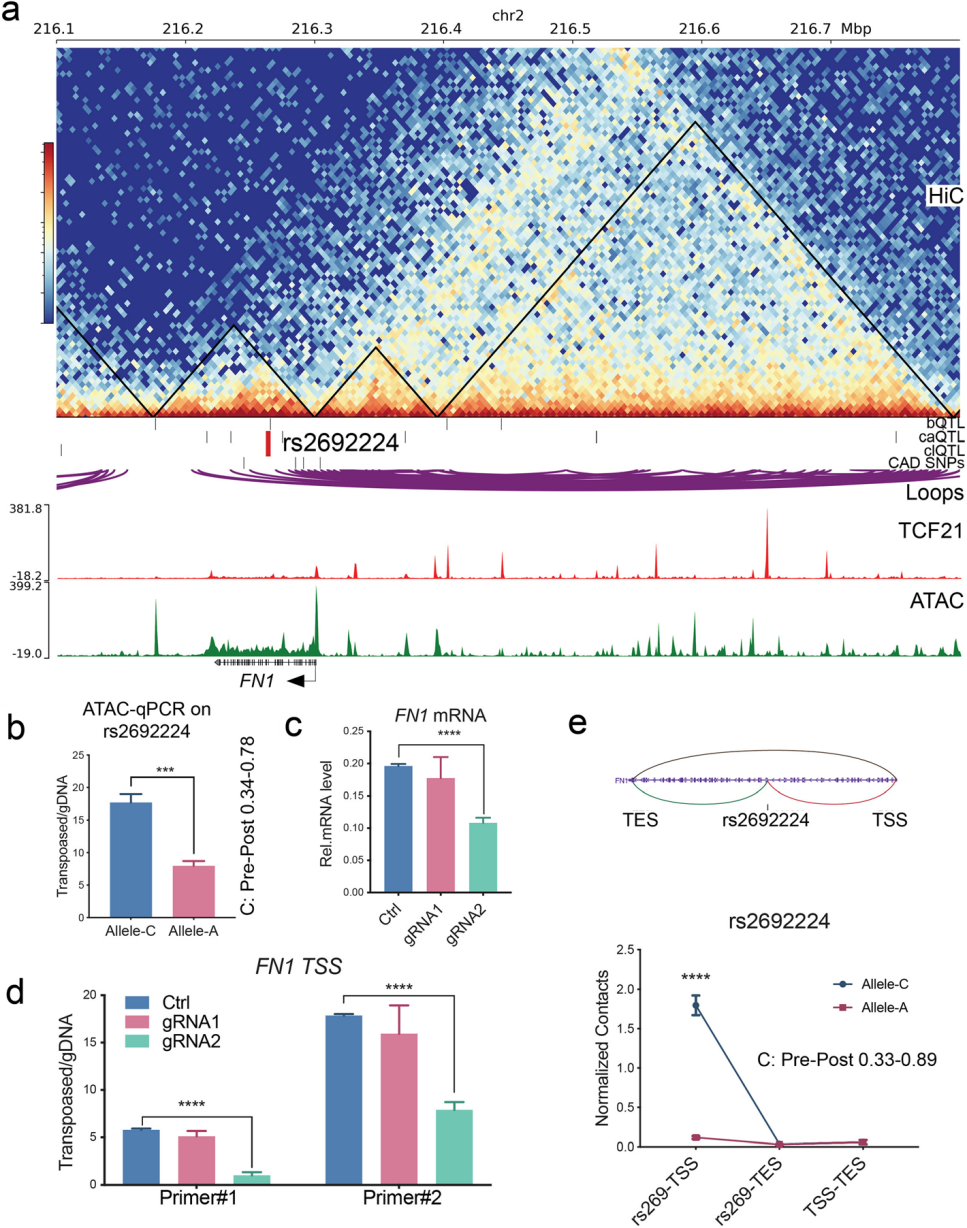
caQTL and clQTL rs2692224 regulate chromatin accessibility and chromosomal interaction at CAD gene *FN1*. **a***FN1* locus, showing caQTL and clQTL rs2692224. **b** Bar graphs show allele-specific enrichment of chromatin accessibility at rs2692224 as identified by allele-specific qPCR. **c** mRNA levels and **d** chromatin accessibility at the TSS region of *FN1* were suppressed by CRISPRi-KRAB targeting at rs2692224. **e** Diagram (top) shows the chromosomal loops between rs2692224 and TSS/TES of *FN1*. 3C-PCR (bottom) indicates the differential chromosomal contacts from rs2692224 to TSS of *FN1*. Pre-post: allele frequencies in the QTL regression data. Shown are means ± SD; *n* = 3; ****P < 0.0001; ***P < 0.001; **P < 0.01; *P < 0.05

With a similar approach, we validated the caQTL and clQTL rs2692224 variant located ~ 40 kb downstream of the *FN1* TSS (Fig. 6a). Combined ATAC-qPCR (Fig. 6b), CRISPRi (Fig. 6c, d), and 3C-PCR (Fig. 6e) data demonstrated that rs2692224 contacts the *FN1* TSS region and allele C has higher chromatin accessibility and chromosomal interaction. Moreover, we further verified the clQTL rs546512774, bQTL, and caQTL rs1037169 located in *ARNTL* locus (Additional file 1: Suppl Fig. 6a-d); clQTL rs1291356, bQTL, and caQTL rs7979663 located in *EMP1* locus (Additional file 1: Suppl Fig. 6e-i); and caQTL, clQTL rs993767, and rs3114275 located in *CCBE1* locus (Additional file 1: Suppl Fig. 6j-l). All of these QTLs were validated with expected directionalities that are consistent with the pre-post frequencies in QTL regression results.

### Allele-specific looping at the CAD gene *FN1* is regulated by TGFβ1 via response clQTL rs2692224

**Fig. 7:**
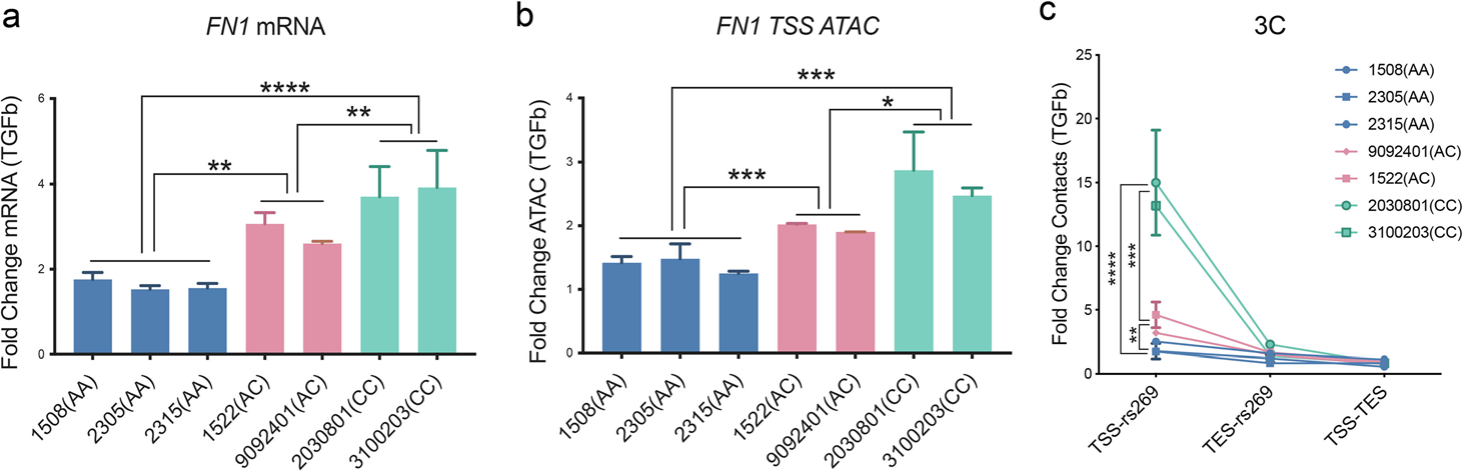
caQTL rs2692224 regulates the allele-specific response of *FN1* to TGFβ signaling. HCASMC lines with different rs2692224 genotypes show distinct activation levels of **a** mRNA expression, **b** chromatin accessibility at the TSS region, and **c** chromosomal contacts between rs2692224 and TSS of *FN1*, in response to TGFβ stimulation. Shown are means ± SD; *n* = 3; ****P < 0.0001; ***P < 0.001; **P < 0.01; *P < 0.05

Fibronectin 1 (FN1) binds to multiple extracellular matrix proteins such as integrin, collagen, and fibrin [34]. It regulates cardiovascular development and responds to TGFβ [28, 35, 36]. Given that TGFβ1 and its primary nuclear signaling molecule SMAD3 are both putative CAD-associated genes [24], we sought to investigate a causal mechanism by which this disease-related signaling pathway could be linked to *FN1* disease association, through the function of QTLs identified in these studies. As described above, inhibiting the region surrounding rs2692224 with CRISPRi leads to the repression of *FN1* expression. We used TGFβ1 to stimulate multiple HCASMC lines which have different genotypes for rs2692224, including three lines with open allele homozygous C|C, three lines with closed allele homozygous A|A, and two heterozygous A|C lines. We first evaluated *FN1* mRNA levels and found that the increase in expression with TGFβ1 stimulation in the cell lines with the C allele was significantly higher than that with the A allele (Fig. 7a). Similarly, the chromatin accessibility responses to TGFβ at this locus were greater with the rs2692224 C alleles (Fig. 7b). In addition, the 3C-PCR results showed that chromosomal interactions are more active in the cell lines containing the C allele compared with the lines containing the A allele (Fig. 7c). Overall, the C|C homozygous HCASMCs are most sensitive to TGFβ1 and A|C heterozygous are less sensitive while A|A homozygous are most insensitive to *FN1* expression, chromatin accessibility, and chromosomal interaction. These functional studies provide significant evidence that variant rs2692224 serves to link the transcriptional response of the CAD-associated gene *FN1* to stimulation by the disease-associated TGFβ1 pathway, through mechanisms that regulate chromatin accessibility and chromosomal interactions.

## Discussion

Recent GWAS have associated thousands of regions of the genome with numerous human traits and complex diseases. For the most part, these associations are likely mediated by variation of gene expression in these loci, due to differences in allelic variation that affects transcription in *cis*. The GWAS data and related insights into the mechanisms of association that are arising from such findings have spurred considerable interest in related mechanisms of transcriptional regulation [37]. Variants that regulate gene expression, eQTLs, have been the most thoroughly investigated, in cell lines and disease-related tissues, and have accelerated the identification of causal genes in associated loci [7, 38–41]. The GTEx consortium efforts have contributed extensively to these efforts, identifying in particular tissue-specific eQTLs [6]. Mapping of QTLs that regulate genomic molecular phenotypes that likely are responsible for eQTL effects on gene expression, such as caQTLs [9] and clQTLs [42], and histone modification, hQTLs [43], has been more limited and restricted primarily to collections of immortalized lymphoblastoid cell lines (LCLs). Studies reported here have significantly expanded our understanding of how these features of the genome regulate gene expression and how perturbation of DNA sequences that determine these features might contribute to variation in risk for complex human diseases.

Because of our interest in the genetic basis of CAD, and in particular the role of vascular smooth muscle cells in this regard, we have undertaken mapping of genomic regulatory variation in primary cultured HCASMC. Mapping of QTLs in LCLs for instance did not show enrichment in CAD-associated loci, underscoring the importance of studies such as these in disease-relevant cell types and tissues [43]. This work was made possible through the pooling approach that dramatically decreases the amount of cell culture work and the expense of multiple assays and sequencing reactions [9, 13]. The individual ChIP-PCR, ATAC-PCR, and 3C-PCR assays that we performed represent the most extensive validation of this approach. QTLs of the CAD-associated transcription factor TCF21 were of particular interest because of the enrichment of its binding in other CAD loci [27] and contribution to the risk at these loci where it modulates the epigenome and thus expression of causal genes [16, 28]. We have previously shown that CAD-associated variants are enriched in HCASMC regions of open chromatin [24] and that these cells contribute a significant portion of CAD risk [15]. We have shown previously that chromosomal looping at CAD loci is regulated by associated allelic variation [17] and mapping of HCASMC clQTLs significantly expands this work with identification of specific variants that are candidate regulators of this genomic feature. To the best of our knowledge, this is the first report of mapping clQTLs in primary cultured human cells.

Investigation of the overlap of different classes of QTLs in HCASMC is informative. In general, the overlap of individual QTLs was modest and likely reflects a number of features of the individual datasets. Given the limited number of subjects in these studies, it is unlikely that we were able to identify the majority of each type of QTL, which would limit the overlap of the individual datasets. Also, analyses for each QTL likely showed variation in the individual SNP that might have been chosen from among a number of variants in linkage disequilibrium in an associated locus. This was demonstrated in our data, as there was significantly increased colocation of bQTLs and caQTLs when variants with linkage disequilibrium (*R*^2^ > 0.8) were considered (Fig. 4b), which was consistent with colocation of these QTLs across the genome (Fig. 4c). Interestingly, both of these classes of variants were also noted to be enriched at JUN binding sites, consistent with a pioneer function for the AP-1 complex (Fig. 3h) [16]. Further, enrichment of these QTLs with the smooth muscle cell MYOCD-SRF complex, SRF binding CArG boxes, indicated a role for these regulatory variants in SMC processes (Fig. 3i). Gene Ontology analysis of genes located nearby to QTLs showed high-level enrichment for terms related to SMC processes such as matrix organization, cell-cell interactions–adherence junctions, and vascular development and angiogenesis. By comparison, clQTLs had less colocation and a different set of enrichment categories, suggesting an independent mechanism from bQTLs and caQTLs.

TF motifs nearby to the bQTLs and clQTLs indicate interactions between the binding and function of these two types of regulatory activities. As expected, there is primarily enrichment for TCF21 and its class I bHLH dimerization partner TCF12 nearby to the TCF21 bQTLs. Enrichment for AP-1 motifs was identified for both the bQTLs and caQTLs, consistent with its known role in HCASMC [16] and its relationship to lineage determining factors in multiple other cell types [44–46]. TEAD motifs were represented nearby to the bQTL and caQTL regions, and these factors are known to play a significant role in SMC lineage determination and disease risk [47]. Further, caQTLs were associated with SMAD3, a known potent regulator of blood vessel development [48–50] and SMC function [51, 52] as well as CAD association [24, 28]. Interestingly, analysis of the closed caQTLs showed low-level enrichment for motifs that mediate binding of ZEB factors, which are known to promote closure of DNA [26].

Perhaps most importantly, mapping these data provides highly useful information for developing credible sets of variants in disease loci implicated by GWAS. For those QTLs that overlap, especially for overlapping bQTLs and caQTLs, there is an increased likelihood that these variants are the active regulatory variant in the region, and thus more likely to contribute to the risk association in the associated locus. However, these QTLs suffer from the same ambiguity regarding functional identity as all variation mapped at a genome-wide level; it is often difficult to discern the functional versus the correlated variants in a haplotype block. In the *CDH13* and *FN1* loci where we have validated the identified QTLs, these QTLs are not in LD with the reported lead SNPs and thus to contribute to disease association would have to be identifying another allele not reported in the GWAS data. The average number of alleles for each GWAS locus is 2–3, and nearby independent alleles are often not reported, so this is a distinct possibility. To link the *FN1* locus QTLs with CAD, we showed that stimulation of HCASMC by TGFβ, a known causal CAD signaling pathway [24, 28, 53], produces a change in the looping pattern as predicted by the clQTL. These molecular QTLs will provide support to fine mapping efforts aimed at identifying disease causal variation, limiting credible sets of genes identified by other methods, and better understanding of the molecular functions of regulatory variation in the human genome.

## Conclusions

In experiments described here, we have used a pooling approach with primary cultured HCASMC to map regulatory variation that mediates binding of the CAD-associated transcription factor TCF21 (bQTLs) with ChIPseq studies, mapped variation that regulates chromatin accessibility (caQTLs) with ATACseq studies, and chromosomal looping (clQTLs) with Hi-C methods. We showed that these QTLs are highly associated with CAD GWAS loci and correlated to lead SNPs in these loci, colocalize with smooth muscle cell transcription factor binding and epigenetic modification, and show allele-specific function in CAD GWAS loci and that these functional mapped variants can serve as response QTLs that link expression of causal CAD genes to epigenetic stimulation. Together, these studies represent the most thorough mapping of multiple QTL types in a highly disease-relevant primary cultured cell type and provide novel insights into their functional overlap and mechanisms that underlie these genomic features and their relationship to disease risk.

## Methods

### Primary cell culture and reagents

Primary human coronary artery smooth muscle cells (HCASMCs) derived from normal human donor hearts were purchased from three different manufacturers, Lonza (CC-2583, *n* = 5), PromoCell (C-12511, *n* = 25), Lifeline Cell Tech (FC-0031, *n* = 4), ATCC (PCS-100-021, *n* = 3) and Cell Applications (350-05a, 35) at passage 2 and were maintained in hEGF, insulin, hFGF-b, and 5% FBS supplemented smooth muscle basal media (Lonza # CC-3182) according to the manufacturer’s instructions. All lines were authenticated by the manufacturer and in the author’s laboratory by morphology and gene expression signature. The detailed samples and subject information can be found in Additional file 4: Suppl Table 3. All experiments were performed on HCASMCs between passages 4 and 6. Purified rabbit polyclonal antibody against human TCF21 (HPA013189) was purchased from Sigma. Recombinant human TGFβ (AF-100-21C) was purchased from PeproTech and was used at 25 ng/mL for 12 h after 24-h serum starvation of the cells. Due to difficulties culturing some of the HCASMC lines, there was slight variation regarding which lines could be included in different QTL studies. We were finally able to pool 65 lines for TCF21 ChIPseq and Hi-C and 71 lines for ATACseq experiments from total 72 lines at passages 4–6.

### ChIP

Briefly, approximately 2 × 10^7^ pooled HCASMC cells were fixed with 1% formaldehyde and quenched by glycine. The cells were washed three times with PBS and then harvested in ChIP lysis buffer (50 mM Tris-HCl, pH 8, 5 mM EDTA, 0.5% SDS). Crosslinked chromatin was sheared for 3 × 1 min by sonication (Branson SFX250 Sonifier) before extensive centrifugation. Four volumes of ChIP dilution buffer (20 mM Tris-HCl, pH 8.0, 150 mM NaCl, 2 mM EDTA, 1% Triton X-100) was added to the supernatant. The resulting lysate was then incubated with Dynabeads™ Protein G (Thermo Scientific, 10009D) and antibodies at 4 °C overnight. Beads were washed once with buffer 1 (20 mM Tris pH 8, 2 mM EDTA, 150 mM NaCl, 1% Triton X100, 0.1% SDS), once with buffer 2 (10 mM Tris pH 8, 1 mM EDTA, 500 mM NaCl, 1% Triton X100, 0.1% SDS), once with buffer 3 (10 mM Tris pH 8, 1 mM EDTA, 250 mM LiCl, 1% NP40, 1% sodium deoxycholate monohydrate), and twice with TE buffer. DNA was eluted by ChIP elution buffer (0.1 M NaHCO3, 1% SDS, 20 μg/mL proteinase K). The elution was incubated at 65 °C overnight, and DNA was extracted with DNA purification kit (Zymo D4013). Library was sequenced on the Illumina HiSeq X instrument to 400 million 150-bp paired-end reads.

### ATAC

Approximately 5 × 10^4^ pooled fresh HCAMSC cells were collected by centrifugation at 500 rcf and washed twice with cold PBS. Nucleus-enriched fractions were extracted with cold resuspension buffer (0.1% NP-40, 0.1% Tween-20, and 0.01% Digitonin) and washed out with 1 mL of cold resuspension buffer containing 0.1% Tween-20 only. Nucleus pellets were collected by centrifugation and resuspended with transposition reaction buffer containing Tn5 transposases (Illumina Nextera TDE1). Transposition reactions were incubated at 37 °C for 30 min, followed by DNA purification using the DNA Clean-up and Concentration kit (Zymo D4013). Libraries were amplified using Nextera barcodes and High-Fidelity polymerase (NEB M0541S) and purified using Agencourt Ampure XP beads (Beckman Coulter A63880) double-size selection (0.5X:0.9X). For qPCR experiments, transposed samples were normalized by genomic DNA which was extracted using Quick-DNA Microprep Kit (Zymo D3020). Library was sequenced on the Illumina HiSeq X instrument to 400 million 150-bp paired-end reads.

### Hi-C

The Hi-C protocol was modified from HiChIP with excluding the antibody purification [54]. Briefly, approximately 97.5M HCASMCs, 1.5M from 65 different cell lines, were pooled and fixed with 1% formaldehyde and quenched by glycine. The cells were washed with PBS and then harvested in Hi-C lysis buffer (10 mM Tris-HCl, pH 8, 10 mM NaCl, 0.2% NP-40). Nucleus pellet was incubated in 0.5% SDS at 62 °C for 10 min and then quenched by water and 10% Triton X-100. MboI (NEB, R0147) enzyme digested DNA for 2 h at 37 °C. Biotin-labeled dATP (Thermo,19524016) and Klenow (NEB, M0210) were used to fill restriction fragment overhangs. The DNA was re-ligated by T4 DNA ligase (NEB, M0202) at room temperature for 4 h. Re-ligated nuclei were lysed by 0.5% SDS and sheared for 2 min by sonication before extensive centrifugation. Supernatant was incubated at 65 °C overnight with Protease K and the DNA was purified. For library construction, set aside DNA by 150 ng to the biotin capture step. Biotin-labeled DNA was pre-bound to Streptavidin C-1 beads (Thermo, 65-001) in biotin binding buffer. After Tween buffer and TD buffer washing, Tn5 transposition was performed on beads at 55 °C for 10 min. Libraries were generated by PCR amplification with Nextera adapters added after washing the beads. Samples were size selected by PAGE purification (300–700 bp) for effective paired-end sequencing and adapter dimer removal. All libraries were sequenced on the Illumina HiSeq 4000 instrument to total 800 million 75-bp paired-end reads.

### Genotyping and phasing

HCASMC genomic DNA was isolated using DNeasy Blood & Tissue Kit (QIAGEN 69506) and quantified using NanoDrop 1000 Spectrophotometer (Thermo Fisher). Macrogen performed library preparation using Illumina’s TruSeq DNA PCR-Free Library Preparation Kit and 150 bp paired-end sequencing on Illumina HiSeq 4000 instrument.

Whole-genome sequencing data were processed with the *GATK* best practice pipeline [15]. *cutadapt* trimmed reads were aligned to the hg19 reference genome with *bwa*. Duplicate reads in alignment result were marked with *picard markduplicate*. We performed indel realignment and base recalibration with *GATK*. The *GATK HaplotypeCaller* was used to generate gVCF files, which were fed into *GenotypeGVCFs* for joint genotype calling. We recalibrated variants using the *GATK VariantRecalibrato*r module. We further phased our call set with *Beagle*. We first used the *Beagle conform-gt* module to correct any reference genotypes if they are different from hg19. We then phased and imputed against 1000 Genomes phase 3 version 5a. Variants with imputation allelic *r*^2^ less than 0.8 and *Hardy*–*Weinberg equilibrium P* value less than 1 × 10^−6^ were filtered out.

### Pooled sequencing data processing

Quality control of pooled ChIPseq data was performed using *fastqc*, and then low-quality bases and adaptor contamination were trimmed by *cutadapt*. Filtered reads were mapped to hg19 genome using *bwa mem* algorithm. Duplicate reads were marked by *picard markduplicate* module and removed with unmapped reads by *samtools*. *macs2 callpeak* was used for peak calling and input as control. Similar approaches were used for pooled ATACseq with the following modifications. We used *bowtie2* to align reads to hg19 genome. *bedtools* was used for reads format converting, and *awk* was used for Tn5 shifting. *macs2 callpeak* with *--broad* was used for peak calling with lambda background*.*

Pooled Hi-C paired-end reads were aligned to the HCASMC phasing data masked hg19 genome using the *HiC-Pro* pipeline. Default settings were used to remove duplicate reads, assign reads to MboI restriction fragments, filter for valid interactions, and generate binned interaction matrices. Aligned reads were assigned to a specific allele on the basis of phasing data. *HiC-Pr*o filtered reads were then processed using the *hicpro2juicebox* and *hicpro2fithic* functions. *FitHiC* and *HiCCUPS* were used to identify high-confidence loops using default parameters (FDR < 1%). The Hi-C matrix file was further converted to h5 format by the *HiCExplorer hicConvertFormat* module. Genome bias of the matrix was corrected by *hicCorrectMatrix*, and *hicFindTADs* was employed to find transcription activation domain (TAD).

### Mapping and analyzing QTLs

Because the genome indices of aligners only include the reference alleles, the reads containing reference alleles rather than alternative alleles have a better chance to be aligned [13, 17], which causes the reference alleles to have higher frequencies than the alternative alleles. We employed the *hornet* (https://github.com/TheFraserLab/Hornet) pipeline to remove this bias in the processed alignments of pooled ChIPseq, ATACseq, and Hi-C reads. HCASMC phasing data were combined with 1000 Genome data of CEU population, filtered by MAF > 2.5%. Mapped reads that overlap the combined SNPs were identified. For each read that overlaps a SNP, its genotype was swapped with that of the other alleles and the read was re-mapped. Re-mapped reads that fail to map to exactly the same location in the genome were discarded.

Pre- and post-allele frequencies and the resulting *P* values were calculated using published pipeline *cisVar* (https://github.com/TheFraserLab/cisVar). This regression-based approach uses post-allele frequencies together with genotypes of each sample to infer the proportion of each sample in the pool [9, 13]. These proportions will be weighted by any genome-wide differences, since these will be naturally incorporated into the post-frequencies used as input to the regression. In this way, pre-allele frequencies already account for some types of trans-acting variation, increasing the power for mapping *cis*-acting differences. For example, if one cell line has a greater abundance of TCF21, leading to widespread increased TCF21 binding, then its alleles will be overrepresented in the post-ChIP reads. By estimating the pre-ChIP allele frequencies directly from the post-ChIP reads, this variation in TCF21 abundance is accounted for, since it will affect the pre- and post-ChIP allele frequencies equally [13]. The *P* value cutoff was determined by a comparison between the output and a previously reported caQTLs dataset using a python script (https://github.com/TheFraserLab/enrich_pvalues) [9, 13].

### GWAS overlap

HCAMSC eQTL data came from a genome-wide association of gene expression with imputed common variation identified in 52 HCASMC lines [15] . CARDIoGRAMplusC4D variant data was from 1000 Genomes-based GWAS meta-analysis [19]. GWAS catalog data came from NHGRI-EBI [18]. The information of these analyzed loci can be found in Additional file 5: Suppl Table 4. Direct overlap of QTLs and GWAS Catalog SNPs was performed with *bed2GwasCatalogBinomialMod1Ggplot* script from *gwasanalytics* package. The calculation criteria of this script were described previously [55]. We used *LDDirection* (https://github.com/MikeDacre/LDDirection), which employs *plink* and 1000 Genome phasing data of CEU population, to calculate the linkage disequilibrium (LD) between QTL and GWAS SNP pairs within 100 kb maximum distance.

### Motif analysis

We used *HOMER findMotifsGenome.pl* script to search for known motifs and to generate de novo motifs among the significant QTLs compared to the non-significant QTLs in ± 50-bp windows with FDR 0.1% cutoff. These results were further validated by *MEME suite*. The known motifs were scanned by *CentriMo* using the JASPAR database while the de novo motifs were analyzed by MEME with score > 5 and *E* value < 10 cutoffs. We also searched the motifs that were differentially enriched among the high-chromatin accessibility or TCF21 binding (open) alleles compared to the low-chromatin accessibility or TCF21 binding (closed) alleles in the shared QTLs. The open allele plus 20 bp on each side (40 bp total) was used as input to *HOMER findMotifs.pl* script, with the closed alleles of the same QTLs used as the background comparison set, or on the opposite direction. Therefore, all significant enrichments are due to QTL variants within the motifs themselves (since any motifs flanking the QTLs would be present in both comparison sets). *PWMScan* was used for position weight matrix scan. We obtained AHR:ARNT (MA0006.1) motif from JASPAR. The JUN and SRF peaks came from the published data [24, 56]. For the differential enrichment tests, we used the non-significant QTLs (*P* > 0.5) to compare their densities on the TF binding sites or open chromatin regions with those of bQTL, caQTL, and clQTL or to compare the ATAC, H3K27ac, and H3K4me1 enrichment levels between these non-significant QTLs and bQTL, caQTL, or clQTL, in ±5-kb windows. We also compared the ATAC, H3K27ac, and H3K4me1 enrichment levels between the anchor regions and their adjacent regions (up to ± 20 kb).

### *Cis*-regulatory functional enrichment and network analysis

We used our Hi-C chromatin interaction data to assign the QTLs to their target genes. The gene and the QTL which were either located in the same anchor of a chromosomal loop or overlapped with the two anchors of a loop were considered as a potential QTL-gene regulatory pair. Gene ontology analysis was done with the PANTHER database. Pathways, biological processes, cellular component, pathways, and disease enrichments were carried out using *Fisher’s exact test* and corrected by FDR 5%.

### Data visualization

ChIPseq and ATACseq bigWig tracks were converted from filtered alignments using *bedtools* and *UCSC utilities*. Hi-C matrix generated by *hicpro2juicebox* was visualized by *Juciebox*. The *HiCCUPS* and *FitHiC* output were converted to bigInteract format by custom *awk* command lines. ChIA-PET data came from ENCODE database (ENCSR000CAD and ENCSR361AYD). Genome browser sessions were generated by *WashU Epigenome Browser* or *pyGenomeTracks*.

### CRISPRi validation

We used dCas9 fused to KRAB to knockdown the enhancer region where candidate SNPs were located. The gRNAs targeting SNPs within the maximum 100 bp distance were designed using *Benching* online tools. Synthesized oligos were then cloned into pLV lentivirus vector containing dCas9-KRAB. For virus production, 8.5 × 10^5^ HEK293T cells were plated in 6-well plate per well. The following day, plasmid encoding lentivirus was co-transfected with pMD2.G and pCMV-dR8.91 into the cells using Lipofectamine 3000 (Thermo Fisher, L3000015) according to the manufacturer’s instructions. ViralBoost Reagent (AlStem Cell Advancements, VB100) was added (1:500) when fresh media were added. Supernatant containing viral particles was collected 72 h after transfection and filtered. To knockdown, we plated 5 × 10^4^ HACSMC cells in 6-well plate per well and cultured for 14 h and then added the virus supernatant to HCASMC for 12 h with 8 μg/mL polybrene. The cells were cultured for an additional 48 h for further experiments with medium change.

### RNA isolation

RNA for all samples was extracted using the RNeasy mini kit (Qiagen 74106). HCASMC RNA (500 ng) were reverse transcribed using the high capacity RNA-to-cDNA Synthesis kit (Applied Biosystems 4387406).

### Quantitative and genotyping PCR

The purified cDNA or dsDNA samples were assayed by quantitative PCR with ABI ViiA 7 and Power SYBR Green Master Mix (ABI 4368706) using custom designed primers (Additional file 6: Suppl Table 5). ChIP samples were normalized by input, ATAC transposed samples were normalized by genomic DNA which was extracted using Quick-DNA Microprep Kit (Zymo D3020), and 3C libraries were normalized by post-ligation whole-genome DNA without biotin purification. Heterozygous genotypes at the candidate loci were determined using TaqMan SNP genotyping qPCR assays (Thermo Fisher Scientific C___2110683_10, C___8714882_10, C___2042333_10, C__27515710_10, C__29302709_10, C__31103265_10, C___3202579_10). Assays were repeated at least three times. Data shown were average values ± SD of representative experiments. The FDR cutoff was 1%.

### Statistical analysis

All experiments were performed by the investigators blinded to the treatments or conditions during the data collection and analysis, using at least two independent biological replicates and treatments/conditions in technical triplicate. R or GraphPad Prism was used for statistical analysis. For motifs and gene enrichment analyses, we used the *cumulative binomial distribution test*. For comparisons between two groups of equal sample size (and assuming equal variance), an unpaired two-tailed *Student’s t test* was performed or in cases of unequal sample sizes or variance a *Welch’s unequal variances t test* was performed. *P* values < 0.05 were considered statistically significant. For multiple comparison testing, one-way analysis of variance (*ANOVA*) accompanied by *Tukey’s* post hoc *test* was used as appropriate. The FDR cutoff was 5% as default unless otherwise indicated. All error bars represent standard error of the mean (SE). The number of asterisks for the *P* values in the graphs indicates the following: ****P < 0.0001, ***P < 0.001, **P < 0.01, and *P < 0.05.

## Supplementary information

**Additional file 1.** Supplemental figures and related figure legends.

**Additional file 2: Table S1.** Regressions of significant TCF21 binding QTLs, chromatin accessibility QTLs and chromosomal looping QTLs.

**Additional file 3: Table S2.** The candidate CAD associated genes with TCF21 binding, chromatin accessibility and chromosomal looping QTLs loci.

**Additional file 4: Table S3.** The samples source and subject information.

**Additional file 5: Table S4.** The analyzed loci in GWAS catalog and CardiogramplusC4D databases.

**Additional file 6: Table S5.** Customized primers sequences used for real-time qPCR and gRNA sequences.

**Additional file 7.** Review history.

## Data Availability

The TCF21 ChIPseq, ATACseq, Hi-C, and Microarray datasets generated and analyzed for the current study are available in the Gene Expression Omnibus (GEO): GSE141752 [57]. The whole-genome sequencing data were directly deposited to Sequence Read Archive (SRA) [58]. For the reference datasets used in the manuscript: Nagao, M. Coronary disease associated gene TCF21 Inhibits Smooth Muscle Cell Differentiation by Blocking the Myocardin-Serum Response Factor Pathway. SRF ChIPseq. GSE124011. https://www.ncbi.nlm.nih.gov/geo/query/acc.cgi?acc=GSE124011 (2019) [56]. Liu, B. Genetic regulatory mechanisms of smooth muscle cells map to coronary artery disease risk loci. RNAseq and eQTL. GSE113348. https://www.ncbi.nlm.nih.gov/geo/query/acc.cgi?acc=GSE113348 (2018) [15]. Miller, CL. Integrative fine-mapping of regulatory variants and mechanisms at coronary artery disease loci. H3K27ac and H3K4me1 ChIPseq. GSE72696. https://www.ncbi.nlm.nih.gov/geo/query/acc.cgi?acc=GSE72696 (2016) [24]. Sazonova, O. Characterization of TCF21 downstream target regions identifies a transcriptional network linking multiple independent coronary artery disease loci. JUN ChIPseq. GSE61369. https://www.ncbi.nlm.nih.gov/geo/query/acc.cgi?acc=GSE61369 (2014) [27].
