# Supplementary material for: Molecular mechanisms of coronary disease revealed using quantitative trait loci for TCF21 binding, chromatin accessibility, and chromosomal looping
Source: Genome Biol. 2020 Jun 8;21:135. doi: 10.1186/s13059-020-02049-5 (PMC7278146; doi:10.1186/s13059-020-02049-5)
Supplement: Supplementary file 1 — Additional file 1. Supplemental figures and related figure legends. [file 13059_2020_2049_MOESM1_ESM.pdf]

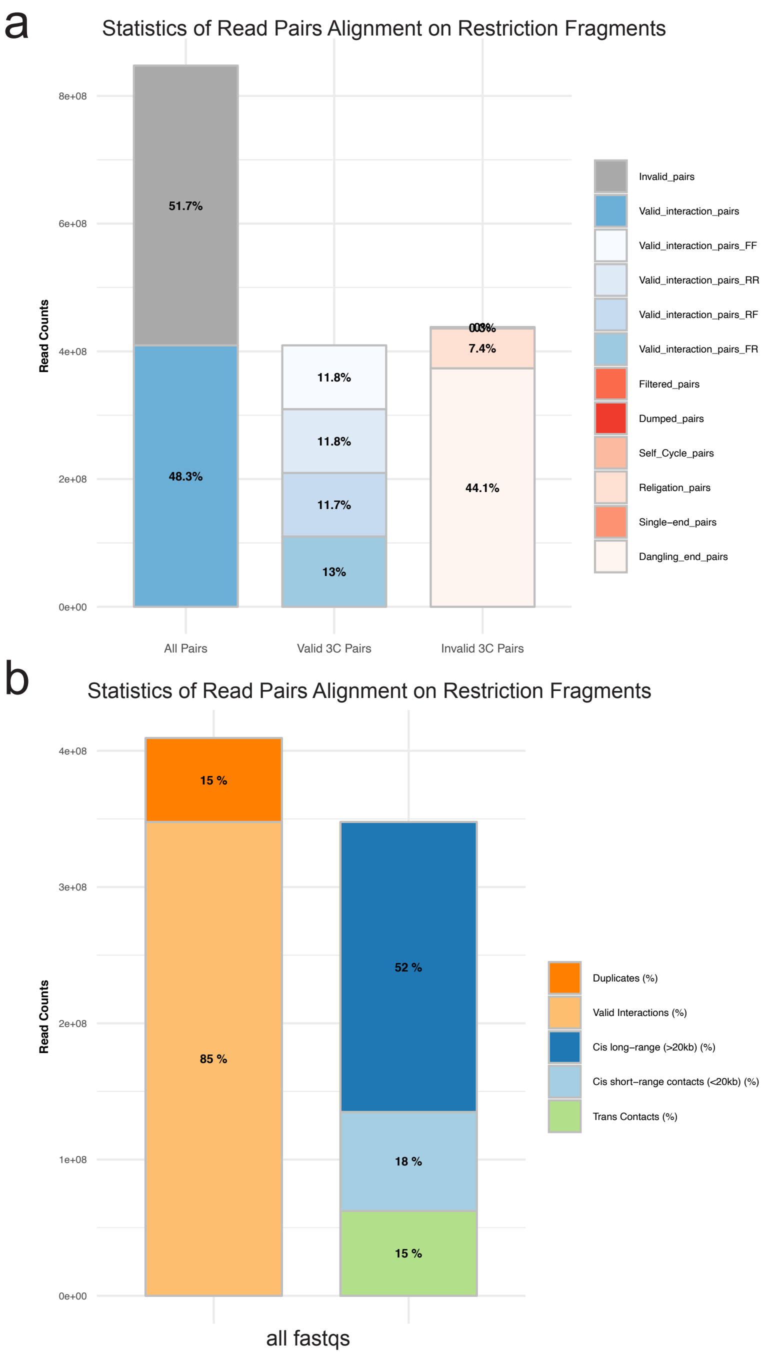

Suppl Figure 1. Quality control of HiC sequencing and interactions.
(a) The valid pairs called from aligned reads and their orientation statistics.
(b) The percentage of valid pairs in all pairs and their classification.

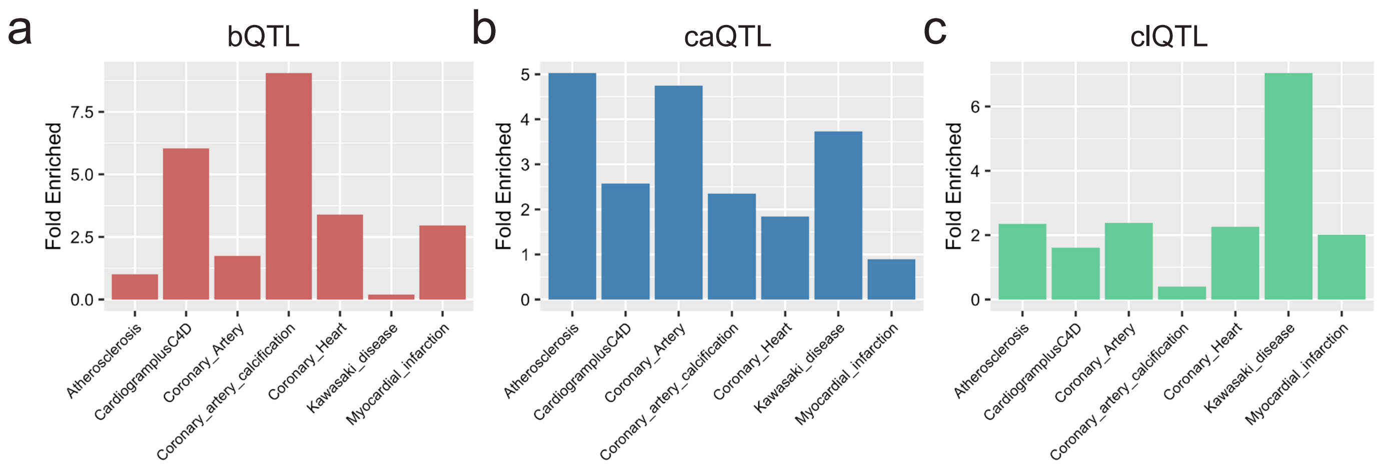

Suppl Figure 2. QTLs are highly associated with GWAS CAD lead SNPs.
(a) bQTL, (b) caQTL and (c) clQTL box plots show the fold enrichment of the ratio of LD R2>0.8 pairs to the total number of QTL-GWAS SNP pairs, for GWAS CAD-associated diseases.

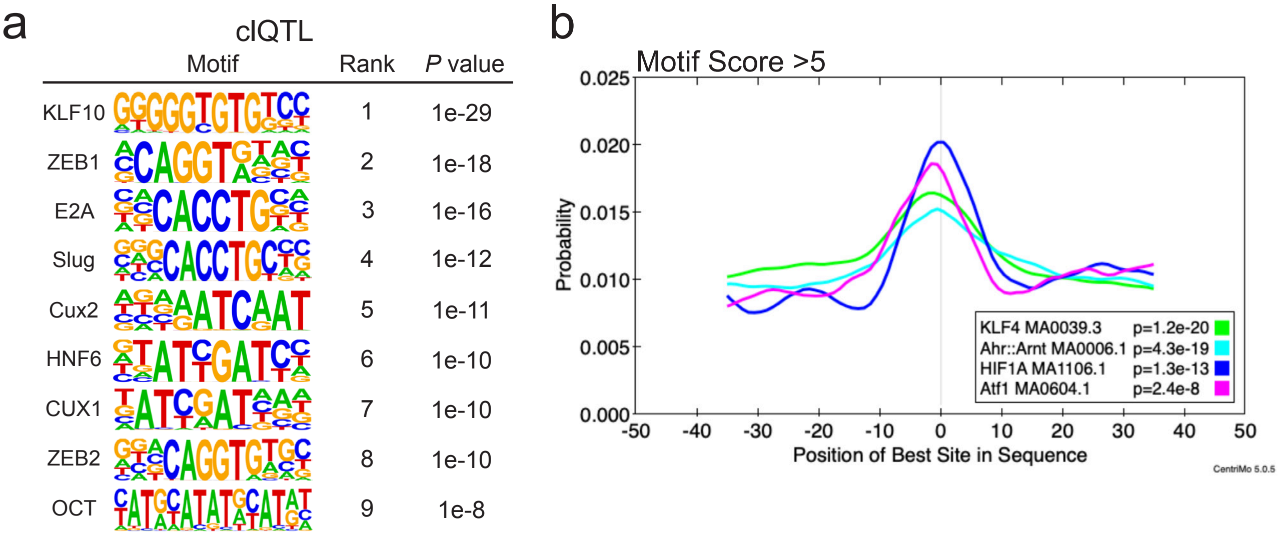

Suppl Figure 3. Motif enrichment analysis at cIQTLs.

(a) HOMER reference motif scanning for ±50bp windows around the cIQTLs. (b) MEME motif analysis of cIQTLs in ±50bp windows with CenrtiMo algorithm.

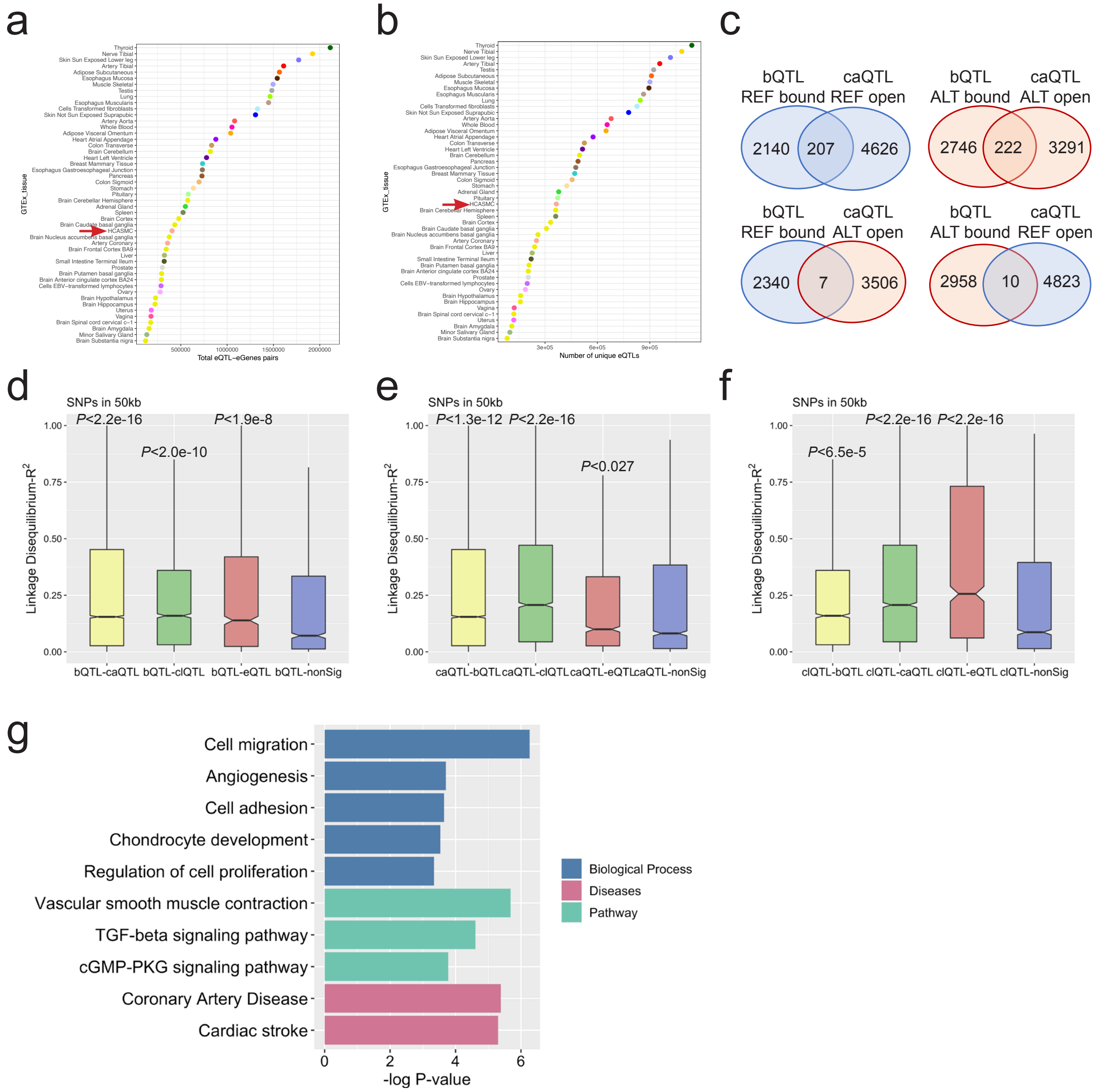

Suppl Figure 4. bQTLs and caQTLs are associated with caQTLs in open chromatin regions. (a) Total eQTL-eGene pairs and (b) total unique eQTL numbers in HCASMC, compared with the GTEx data. (c) Venn diagram shows the directionality of bQTL and caQTL overlap. Top left: bQTL reference (REF) as bound allele overlap with caQTL REF open allele. Top right: bQTL alternative (ALT) as bound overlap with caQTL ALT open allele. Bottom left: bQTL REF as bound, caQTL ALT open allele. Bottom right: bQTL ALT as bound overlap caQTL REF as open. Box plots show linkage disequilibrium (LD) R2 distributions of (d) clQTL, (e) caQTL and (f) bQTL to all other QTL pairs, with non-significant QTLs used as control. (g) Bar graphs show Gene Ontology analysis of the overlapped target genes between bQTL, caQTL and clQTL.

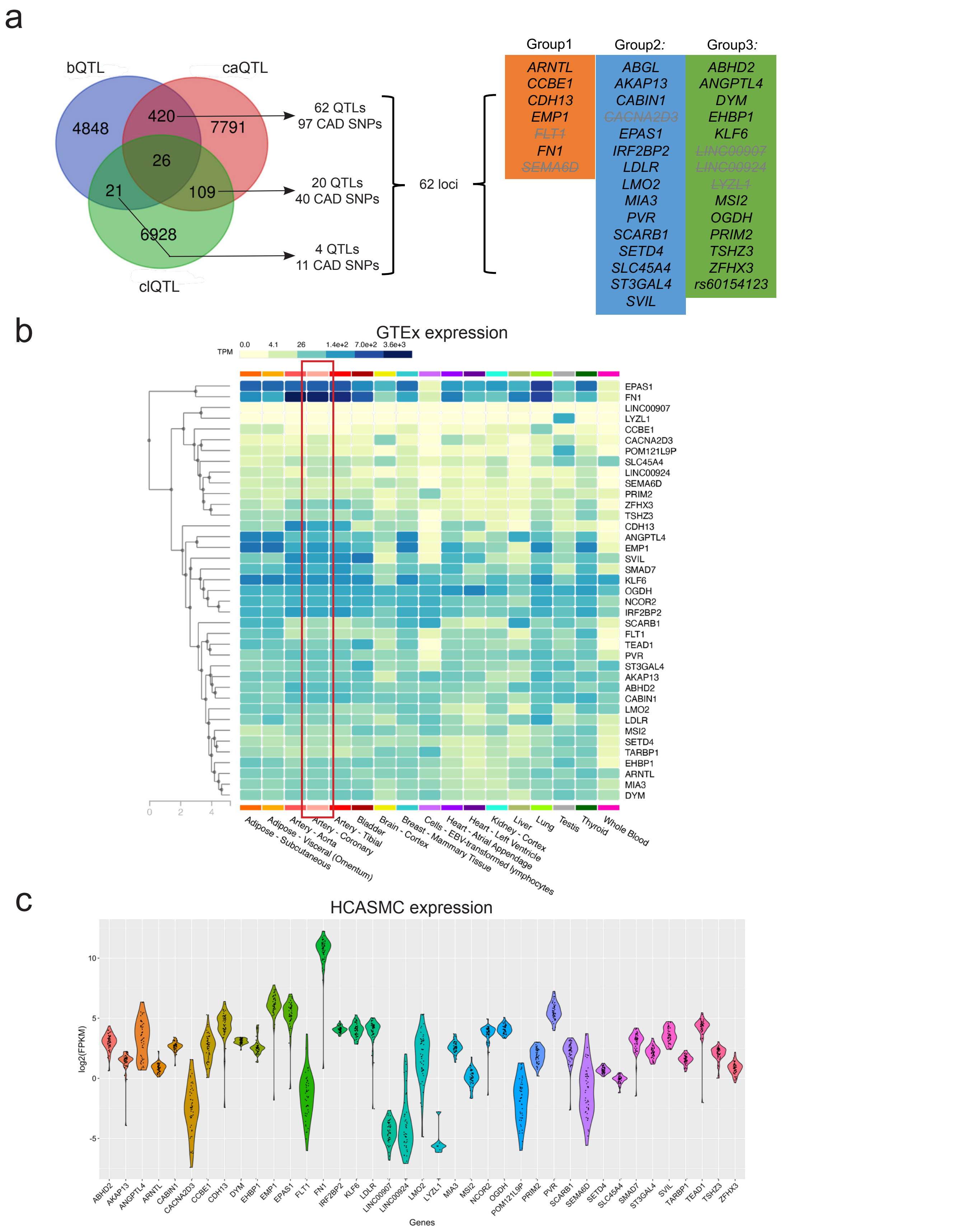

Suppl Figure 5. Evaluation of CAD causal genes located in bQTL, caQTL and clQTL loci.
(a) Screening of CAD causal genes located at directly overlapping bQTL, caQTL and clQTL loci. (b) GTEx and c) RNAseq expression in HCASMC lines showing the expression levels of these CAD causal genes.

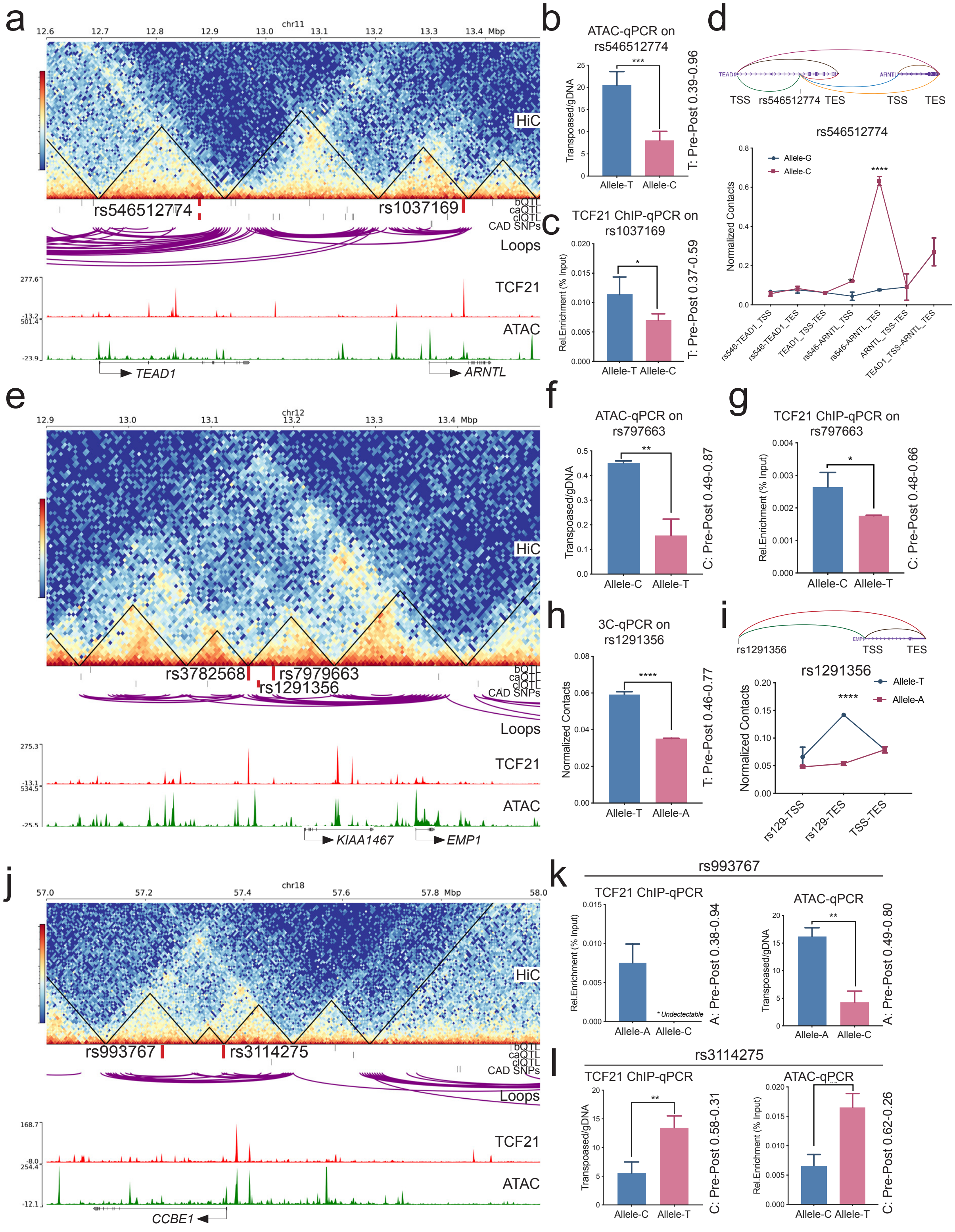

Suppl Figure 6. QTLs located in multiple GWAS CAD loci show allele specific TCF21 binding, chromatin accessibility and chromosomal looping.

(a) ARNTL-TEAD1 locus, showing bQTL rs1037169 and ca/clQTL rs546512774. Bar graphs indicate allelic enrichments of (b) chromatin accessibility and (c) TCF21 binding at rs1037169 identified by allele-specific qPCR. (d) Diagram (top) shows the chromosomal loops between rs546512774 and TSS/TES of ARNTL and TEAD1. 3C-PCR (bottom) showing the differential chromosomal contacts from rs546512774 to TSS and TES of ARNTL. (e) EMP1 locus, showing b/caQTL rs7979663 and clQTL rs1291356. Bar graphs show allelic enrichments of (f) chromatin accessibility, (g) TCF21 binding on rs7979663 and (h) chromosomal contacts at rs1291356 identified by allele-specific qPCR. (i) Diagram (top) shows the chromosomal loops between rs1291356 and TSS/TES of EMP1. 3C-PCR (bottom) showing the differential chromosomal contacts from rs1291356 to TSS and TES of EMP1. (j) CCBE1 locus, showing b/caQTL rs993767 and rs3114275. Bar graphs show allelic enrichments for (k) chromatin accessibility and (l) TCF21 binding on rs993767 and rs3114275 identified by allele-specific qPCR. Pre-post: allele frequencies in the QTL regression data. Shown are means ± SD; n=3; **** P <0.0001; *** P <0.001; ** P <0.01; * P <0.05.
