# Supplementary material for: Molecular mechanisms of coronary disease revealed using quantitative trait loci for TCF21 binding, chromatin accessibility, and chromosomal looping
Source: Genome Biol. 2020 Jun 8;21:135. doi: 10.1186/s13059-020-02049-5 (PMC7278146; doi:10.1186/s13059-020-02049-5)
Supplement: Supplementary file 7 — Additional file 7. Review history. [file 13059_2020_2049_MOESM7_ESM.docx]

Review History

**First round of review**

**Reviewer 1**

**Are you able to assess all statistics in the manuscript, including the appropriateness of statistical tests used?** No, I do not feel adequately qualified to assess the statistics.

**Comments to author:**

Summary: The authors use an elegant, multi-pronged approach to comprehensively identify quantitative trait variants associated with cardiovascular disease. They thoroughly describe the overlap of QTLs from multiple methods, namely TCF21-CHiPseq, chromatin accessibility ATAC-seq, and chromosomal looping. Furthermore, the authors use CRISPRi knockout to validate target loci.

Main Points: These studies will provide a valuable resource for aiding in the discovery of genetic associations with cardiovascular disease.

Minor points- Figure 2 a-c does not indicate what or if there are differences according to the varying colors of the text. Figure 7 lacks statistical p-values that should be added into the figure.

**Reviewer 2**

**Are you able to assess all statistics in the manuscript, including the appropriateness of statistical tests used?** Yes, and I have assessed the statistics in my report.

**Comments to author:**

Zhao et al. mapped quantitative trait loci (QTLs) for ChIP-seq (TCF21, bQTLs), ATAC-seq (caQTLs) and HiC (clQTLs) in human coronary artery vascular smooth muscle cells (HMASMC) to identify molecular mechanisms for coronary artery disease (CAD). The QTLs overlapped between the sequencing methods and were associated with GWAS CAD variants. Functional characterization validated the functional associations between variants and CAD genes.
Rather than standard QTL approaches, the authors used pools of 65-71 samples, sequenced these pools at high read depth (>400M) and compared the observed allele frequency of each variant from ChIP-seq, ATAC-seq and HiC with the expected allele frequency obtained from whole genome sequencing. The authors extracted lead variants from several CAD diseases in the GWAS catalog to compare with the mapped QTLs (within 1kb) and found strong colocalization with all three QTLs types. They next focused on several known CAD loci and determined the mechanisms of associations between QTLs and gene expression by using CRISPRi and 3C.

The abstract is well written but there are several sections of the manuscript that are difficult to follow and understand. At several points in the Results section it is difficult to follow how the analyses are supporting the main take home points of the paper as outlined in the abstract:
*     show significant overlap of the QTLs, and their relationship to smooth muscle specific genes and the binding of smooth muscle transcription factors.
*     use multiple analyses to show that these QTLs are highly associated with CAD GWAS loci and correlated to lead SNPs in these loci where they show allelic effects.
*     Verified with genome editing that identified functional variants can regulate both chromatin accessibility and chromosomal looping, providing new insights into functional mechanism regulating chromatin state and chromosomal structure.
*     Directly link the disease associated TGFβ1-SMAD3 pathway to the CAD associated FN1 gene through a response QTL that modulates both chromatin accessibility and chromosomal looping.

The manuscript could be improved by the authors focusing on how the analyses support these take home messages and emphasizing specific points of high interest. Additionally, the statistical analyses need to be improved, and the methods and results need more details.

Introduction:
Page 3, second paragraph does not really provide the reader useful information to understand the paper.  It appears that the following is referring to QTLs in general and not to pooled eQTL studies.  Referring to QTL studies in general the statements are not true.  Perhaps the authors should help the reader understand the strengths of pooled QTL studies as previously demonstrated.

"Such efforts have provided important information regarding the regulatory structure of the non-coding sequence in the genome and identified how such variation may regulate the risk for complex human disease. However, in general these studies conducted with large high throughput genomic datasets have not been validated with targeted follow-up studies, and the ramifications of their allele-specific effects not investigated. Also, these studies have primarily been conducted in lymphoblastoid cell lines and restricted to a single type of QTL, and thus have not been examined in the context of overlap of QTL functions and their relevance for a broad range of human diseases."

QTL detection:
-     Subject information is missing. A supplemental table is needed with sample and subject data, including source, age, sex, ethnicity (either from the vendor or calculated based on genotypes), clinical history of cardiac disease (if available).
-     Figure 1A: 72 individuals, but the text describes 65 lines for ChIP and Hi-C and 71 for ATAC. Please, explain either in the text or in the figure.
-     Page 4, Line 48: "Our pooled Hi-C loops showed similar patterns but with a higher resolution and more chromosomal contacts compared to the previously reported data." Should reference what previously reported data the authors refer to.
-     Statistical analyses can be improved:
o     Page 5, line 12: "Although the pre-post frequency distributions showed a slight reference allele frequency bias after regression, consistent with previously reported studies [13, 16], the majority of variants showed a balanced pattern". From the figure (Figure 1D-F) it is very hard to see any reference allele frequency bias. Furthermore, what does "after regression" mean? It is not described. The methods describing these analyses need to be improved.
o     Page 5, line 14: why was the p-value cutoff set to 1e-4? What were the number of variants tested?

Colocalization with GWAS:
-     Supporting data for the GWAS analysis is missing:
o     Please, provide a list of the GWAS loci analyzed
o     Please, describe why the 27 randomly selected diseases were selected. Wouldn't it be better to compare with all the other diseases or to find other diseases with similar numbers of GWAS loci?
-     Colocalization refers to a very specific analysis (Giambartolomei, 2014). Please describe what "colocalization" is meant in this study.

Colocalization of QTLs with TF binding and epigenetic modifications:
-     The authors show that clQTLs have enrichment for different TF binding sites than other QTLs (Page 7, line 12): why do they find these differences? One hypothesis that comes to mind is that binding sites enriched at clQTLs are associated with chromatin structure. Can the authors interpret these results?
-     The results described in Figure 3F-H are only qualitative. If the authors can support their observations with statistical tests, their conclusions would be stronger.

Overlap between QTL types:
-     Page 8, line 14: "Our next goal was to characterize loci with bQTL, caQTL and clQTL colocalization" is very vague and does not provide a rationale for the analysis described in the paragraph.
-     Figure 4A-B would be more informative if the number of tested samples and the number of genes analyzed were taken into account (take Figure 1C in the GTEx V.7 paper as example: http://www.nature.com/doifinder/10.1038/nature24277).
-     Interpretation of some of the results is missing:
o     How do the authors explain the observation that "Each QTL group had numerous exact matches with eQTLs while there were few overlaps for all four QTL groups" (page 8, line 24)?
o     In general, why do the authors find so little overlap between different QTL types? It is surprising that only a very small fraction of QTLs is associated with multiple phenotypes.
o     Figure 4C and its corresponding Figure S4D-F are missing statistical tests that show that "the R2 distributions of eQTL to significant (P<10-4) bQTLs, caQTLs or clQTLs was statistically higher than those to non-significant (P>0.5) QTLs" (page 8, line 44)
o     Page 8, line 50: why don't the authors use Hi-C data to find potential target genes? There can be multiple ways to do this, including: finding chromatin loops with their two anchors that respectively overlap or are close to a TSS and a QTL; or QTLs and target genes that are included in the same TAD.

Causal loci analysis:
-     The introductory sentence (page 9, line 15-19) seems to relate to analyses done in the previous sections. Was anything different done here?
-     How were the 36 candidate causal genes selected (page 9, line 24)? A supplemental table describing these loci would be helpful
-     Page 9, line 28: "most of these gene were expressed in both GTEx coronary artery tissues and HCASMC". This sentence is meaningless: are these genes more likely to be expressed in coronary artery and HCASMC than other tissues? Are they expressed at higher levels in general?
-     How were the genes selected for validation (ARNTL, CCBE1, CDH13, EMP1 and FN1)? They are not the most highly expressed (several other genes have higher expression than ARNTL) and all the genes in Figure S5B-C are already known to be in CAD-associated loci.
-     CDH13 validation: the authors use CRISPRi on the clQTL (10 kb downstream of the TSS). In Figure 5A there are two anchors that overlap the variant, with their other anchors falling in introns of CDH13. What is the mechanism by which CDH13 is downregulated when the variant is silenced? The variant is 10 kb downstream of the TSS (so the anchor overlapping the variant is not at the TSS, as Hi-C has 5 kb resolution) and there are no loops connecting the variant to the TSS (if the hypothesis is that the variant overlap a regulatory element for CDH13). Also, why do the authors look for chromatin interactions between the variant and the TSS when the loop affected by the variant does not connect them? Wouldn't it be more informative to find the chromatin interactions between the anchor overlapping the variant, the other anchor (that shares the loop affected by the clQTL) and the TSS?
-     In general, in Figure 5A, 6A and their corresponding supplemental figures, can the authors highlight which loops are affected by the clQTLs?

Discussion:
-     Please, reformulate the sentence "The GWAS data and related insights into the mechanisms of association that are arising from such findings have spurred investigation of the relationships between genetic variation and various genomic features that relate to regulation of transcription". It is very hard to understand its meaning.
-     In several places in the Discussion, the authors compare QTLs from this study to LCL QTLs. GTEx performed eQTLs on ~50 tissues, which demonstrated that many eQTLs are tissue-specific. Please, mention this in the Discussion. The analysis of the overlap between HCASMC and LCL QTLs should be moved to the Results and accompanied by figures/tables, rather than being described in the Discussion.
-     Page 12, line 28: "The finding of colocalization of different classes of QTLs in HCASMC is highly informative". The overlap between different QTL types is very low: how do the authors justify this as highly informative?
-     I could not find a statistical analysis supporting the statement "In our data there was significant colocalization of bQTLs with caQTLs, and both of these with eQTLs" (page 12, line 35).
-     Page 13, line 26: "For those QTLs that overlap, especially for overlapping bQTLs and caQTLs, there is an increased likelihood that these variants are the active regulatory variant in the region, and thus more likely to contribute to the risk association in the associated locus". How was this measured?

Methods:
-     Mapping and analyzing QTLs: how was the sample source (samples were purchased from 3 different sources) taken into account for this analysis? What about other covariates (ethnicity and sex for example)?
-     Page 17, line 52: "In this way, pre-allele frequencies already account for some types of trans-acting variation, increasing the power for mapping cis-acting differences". Please, explain what this sentence means and what is intended by "trans-acting variation".
-     GWAS overlap: without knowing the ethnicity of the individuals, it is impossible to know if LD should be calculated based on the CEU population.
-     Motif analysis: "These results were further validated by MEME". How was this done and where in the results is this described?
-     Statistical analysis: FDR corrections are missing throughout the text.

**Authors Response**

**Point-by-point responses to the reviewers’ comments:**

***Reviewer #1 comments****: Summary: The authors use an elegant, multi-pronged approach to comprehensively identify quantitative trait variants associated with cardiovascular disease. They thoroughly describe the overlap of QTLs from multiple methods, namely TCF21-CHiPseq, chromatin accessibility ATAC-seq, and chromosomal looping. Furthermore, the authors use CRISPRi knockout to validate target loci.
Main Points: These studies will provide a valuable resource for aiding in the discovery of genetic associations with cardiovascular disease.
Minor points- Figure 2 a-c does not indicate what or if there are differences according to the varying colors of the text. Figure 7 lacks statistical p-values that should be added into the figure.*

We thank Reviewer 1 for these positive comments. The colors are for different categories of the diseases. Specifically, cardiovascular related diseases were labeled with “Royal2” palettes by R package “wesanderson”. We now indicate this in figure legends. The P-value significance has now been indicated in Figure 7 (* *P*<0.05, ** *P*<0.01, *** *P*<0.001, **** *P*<0.0001).

***Reviewer #2:*** *Zhao et al. mapped quantitative trait loci (QTLs) for ChIP-seq (TCF21, bQTLs), ATAC-seq (caQTLs) and HiC (clQTLs) in human coronary artery vascular smooth muscle cells (HMASMC) to identify molecular mechanisms for coronary artery disease (CAD). The QTLs overlapped between the sequencing methods and were associated with GWAS CAD variants. Functional characterization validated the functional associations between variants and CAD genes. Rather than standard QTL approaches, the authors used pools of 65-71 samples, sequenced these pools at high read depth (>400M) and compared the observed allele frequency of each variant from ChIP-seq, ATAC-seq and HiC with the expected allele frequency obtained from whole genome sequencing. The authors extracted lead variants from several CAD diseases in the GWAS catalog to compare with the mapped QTLs (within 1kb) and found strong colocalization with all three QTLs types. They next focused on several known CAD loci and determined the mechanisms of associations between QTLs and gene expression by using CRISPRi and 3C.* 

***Comment:*** *The abstract is well written but there are several sections of the manuscript that are difficult to follow and understand. At several points in the Results section it is difficult to follow how the analyses are supporting the main take home points of the paper as outlined in the abstract:
*	show significant overlap of the QTLs, and their relationship to smooth muscle specific genes and the binding of smooth muscle transcription factors.
*	use multiple analyses to show that these QTLs are highly associated with CAD GWAS loci and correlated to lead SNPs in these loci where they show allelic effects. 
*	Verified with genome editing that identified functional variants can regulate both chromatin accessibility and chromosomal looping, providing new insights into functional mechanism regulating chromatin state and chromosomal structure. 
*	Directly link the disease associated TGFβ1-SMAD3 pathway to the CAD associated FN1 gene through a response QTL that modulates both chromatin accessibility and chromosomal looping.*
*The manuscript could be improved by the authors focusing on how the analyses support these take home messages and emphasizing specific points of high interest. Additionally, the statistical analyses need to be improved, and the methods and results need more details.*

We thank the Reviewer for these comments. We have made substantial changes to the text to reflect the direct relevance of the various types of data to the main findings and conclusions of the paper. These are outlined in detail in the following discussion points and broadly throughout the manuscript as indicated in the revised manuscript. We have also extensively improved and increased the statistical analyses of the various types of data, significantly increasing the relevance of the analyses. Regarding the overlap of the QTLs, we have now edited the sentence in the Abstract to be more circumspect as follows, and we have indeed discussed the specific details of the overlap in the Results section, and added a discussion of the limitations of this type of study regarding ascertaining overlap and the implications of our findings in this regard.

Abstract: We examine in detail the overlap of these QTLs, and their relationship to smooth muscle specific genes and the binding of smooth muscle transcription factors.

***Comment:*** *Introduction: Page 3, second paragraph does not really provide the reader useful information to understand the paper. It appears that the following is referring to QTLs in general and not to pooled eQTL studies. Referring to QTL studies in general the statements are not true. Perhaps the authors should help the reader understand the strengths of pooled QTL studies as previously demonstrated.
"Such efforts have provided important information regarding the regulatory structure of the non-coding sequence in the genome and identified how such variation may regulate the risk for complex human disease. However, in general these studies conducted with large high throughput genomic datasets have not been validated with targeted follow-up studies, and the ramifications of their allele-specific effects not investigated. Also, these studies have primarily been conducted in lymphoblastoid cell lines and restricted to a single type of QTL, and thus have not been examined in the context of overlap of QTL functions and their relevance for a broad range of human diseases."* 

We thank the Reviewer for this useful comment. We have now edited the text to reflect the recommendations of the Reviewer and this paragraph now reads as follows:

Page 3, line 23: Introduction

The regulatory variants that modulate other genomic features have also been characterized. QTLs for histone modification and chromatin accessibility, and more recently chromosomal looping have also been mapped [9-12]. Such efforts have provided important information regarding the regulatory structure of the non-coding sequence in the genome and identified how such variation may regulate the risk for complex human disease. In general, these studies have required conducting time consuming and costly assays for each of the large numbers of individuals that is required to reach relevant genome wide levels of statistical association. Recently, we have developed an efficient approach for mapping molecular QTLs that employs sample pooling prior to sequencing, and employed this method to map QTLs for transcription factor binding [13], methylation [14], and chromatin accessibility [9]. This pooling minimizes experimental variability between samples (both within and between experimental ‘batches’), considerably reducing the cost and effort of QTL mapping when compared to standard individual level approaches [13].

***Comment:*** *QTL detection: Subject information is missing. A supplemental table is needed with sample and subject data, including source, age, sex, ethnicity (either from the vendor or calculated based on genotypes), clinical history of cardiac disease (if available).*

We thank the Reviewer for this helpful suggestion. We have now added an additional Supplemental Table 3 (Additional file 4) with sample sources and subject information.

***Comment:*** *Figure 1A: 72 individuals, but the text describes 65 lines for ChIP and Hi-C and 71 for ATAC. Please, explain either in the text or in the figure.*

Due to difficulties culturing some of the HCASMC lines there was slight variation regarding which lines could be included in different QTL studies. We were finally able to pool 65 lines for TCF21 ChIPseq and Hi-C, and 71 lines for ATACseq experiments from total 72 lines at passage 4-6. This text is now included in the Methods section of the manuscript, and the specific cell lines used for each analysis are indicated in Suppl. Table 3.

Page 14, line 52:

Due to difficulties culturing some of the HCASMC lines there was slight variation regarding which lines could be included in different QTL studies. We were finally able to pool 65 lines for TCF21 ChIPseq and Hi-C, and 71 lines for ATACseq experiments from total 72 lines at passage 4-6.

***Comment:*** *Page 4, Line 48: "Our pooled Hi-C loops showed similar patterns but with a higher resolution and more chromosomal contacts compared to the previously reported data." Should reference what previously reported data the authors refer to.*

We were referring to data presented in publication at Nat Genet 2017, 49:1602-1612. We have added this citation to the text.

***Comment:*** *Statistical analyses can be improved: Page 5, line 12: "Although the pre-post frequency distributions showed a slight reference allele frequency bias after regression, consistent with previously reported studies [13, 16], the majority of variants showed a balanced pattern". From the figure (Figure 1D-F) it is very hard to see any reference allele frequency bias. Furthermore, what does "after regression" mean? It is not described. The methods describing these analyses need to be improved.*

Thank you for this suggestion. We rephased this sentence and moved it to Methods with additional explanation for how the reference allele bias was generated. The regression analysis details were reported in our previous study (eLife 2019;8:e39595).

Page 17, line 48:

Because the genome indices of aligners only include the reference alleles, the reads containing reference alleles rather than alternative alleles have a better chance to be aligned [13, 17], which causes the reference alleles to have higher frequencies than the alternative alleles. We employed the *hornet* (https://github.com/TheFraserLab/Hornet) pipeline to remove this bias in the processed alignments of pooled ChIPseq, ATACseq and HiC reads.

Rebuttal Fig 1. Enrichment plot showing the percentage kept of the p-value <0.05 QTLs (left axis), and the enrichment score (right axis, overlapped significant numbers: overlapped non-significant numbers), with p-value cutoffs from 0.05 to 1e-10.

***Comment****: Page 5, line 14: why was the p-value cutoff set to 1e-4? What were the number of variants tested?*

Unfortunately, there was a typo in the original manuscript, the P-value cutoff should be 1e-3(0.001), not 1e-4, and we now have corrected this in the manuscript. The cutoff was based on an enrichment test which compares P-values between our dataset and a previously reported caQTL dataset using a python script (<https://github.com/TheFraserLab/enrich_pvalues>) (eLife 2019;8:e39595) (**see Rebuttal Fig.1).** We also added this information to the Methods section.

Page 18, line 26

The P-value cutoff was determined by a comparison between the output and a previously reported caQTLs dataset using a python script (https://github.com/TheFraserLab/enrich_pvalues) [9, 13]. We have now listed the total number variants tested in the Results section as follows.

Page 4, line 59**:**

With fold enrichment-optimized [9] at *P* value cutoff 10^-3^, we obtained 5315 significant bQTLs, 8346 caQTLs and 7084 clQTLs with the regression analysis from total 60,706, 455,165 and 231,7347 tested variants, respectively (Figs. 1d, 1e and 1f, Additional file 2: Suppl Table. 1).

***Comment:*** *Colocalization with GWAS: Supporting data for the GWAS analysis is missing: Please, provide a list of the GWAS loci analyzed*

We have now provided an additional file 5, Suppl Table 4, with analyzed GWAS loci information.

***Comment****: Please, describe why the 27 randomly selected diseases were selected. Wouldn't it be better to compare with all the other diseases or to find other diseases with similar numbers of GWAS loci?*

The reason we didn’t compare with all other GWAS diseases is because there are 177629 loci in 4294 different diseases in the GWAS catalog (Nucleic Acids Research, 2019, Vol. 47 (Database issue): D1005-D1012.). Specifically, the number of loci in cardiovascular associated diseases are from 45 to 1194. It is hard to show all the disease comparisons with these large numbers of loci. The loci number range of the 27 random selected diseases can actually cover those of the cardiovascular associated diseases **(Rebuttal Fig.2)**

***Comment:*** *Colocalization refers to a very specific analysis (Giambartolomei, 2014). Please describe what "colocalization" is meant in this study.*

Unless otherwise noted, colocalization in the manuscript refers to the same SNPs being represented in two different types of data, primarily QTLs. To avoid confusion, we have changed this term to “colocate” or “overlap” throughout the manuscript.

Rebuttal Fig 2 The loci numbers of the cardiovascular diseases and the random selected comparison diseases in GWAS catalog database.

***Comment:*** *Colocalization of QTLs with TF binding and epigenetic modifications: The authors show that clQTLs have enrichment for different TF binding sites than other QTLs (Page 7, line 12): why do they find these differences? One hypothesis that comes to mind is that binding sites enriched at clQTLs are associated with chromatin structure. Can the authors interpret these results?*

Well, this is indeed a great question. We have discussed this question a great deal among the different authors, and surveyed the literature, but cannot provide an explanation that is supported with meaningful data. We feel that pointing out this fact is one of the more important aspects of our work. Perhaps as the Reviewer speculates, chromatin structure just has a very different regulatory mechanism than chromatin accessibility for instance. We did find enrichment of TFs ZEB1/2 and ARNT, and perhaps these factors have additional roles that we have not yet appreciated in relation to chromatin structure. These factors do interact with p300 and HDACs (J Cancer. 2017; 8(8): 1453–1459. The Journal of Biochemistry, Volume 122, Issue 4, October 1997, Pages 703–710).

***Comment****: The results described in Figure 3F-H are only qualitative. If the authors can support their observations with statistical tests, their conclusions would be stronger.*

We appreciate this recommendation, we have now conducted relevant statistical tests. For Fig.3f, we compared the bQTL, caQTL or clQTL densities with nonsignificant QTL densities on the TCF21, SRF, AHR:ARNT binding sites and ATAC peaks with window size +/-5kb. For Fig3G, we compared the ATAC, H3K27ac and H3K4me1 enrichment levels between bQTL, caQTL or clQTL and nonsignificant QTLs. For Fig.3H, we compared the ATAC, H3K27ac and H3K4me1 enrichment levels on between the anchor regions (yellow highlighted) and their adjacent regions (up to +/-20kb). These P values have been labeled on the figures and we also added the above analyses to the Methods section.

Rebuttal Fig 3. The sample sizes and the QTL/target gene ratios of the TCF21 binding, chromatin accessibility, chromosomal looping, and eQTLs.

***Comment:*** *Overlap between QTL types: Page 8, line 14: "Our next goal was to characterize loci with bQTL, caQTL and clQTL colocalization" is very vague and does not provide a rationale for the analysis described in the paragraph.*

This is a helpful comment, we have added text to help put the work in context, editing the text as follows.

Page 8, line 15:

We hypothesized that QTLs for the different molecular traits would collocate in a number of loci and that these loci would show a greater likelihood of identifying genes and pathways that support specific functions of SMC and their relationship to vascular disease. Our next goal was thus to characterize loci with bQTL, caQTL and clQTL colocation.

***Comment:*** *Figure 4 would be more informative if the number of tested samples and the number of genes analyzed were taken into account (take Figure 1C in the GTEx V.7 paper as example:* [*http://www.nature.com/doifinder/10.1038/nature24277*](http://www.nature.com/doifinder/10.1038/nature24277)*).*

We have taken the Reviewer’s comment and created the suggested graph **(Rebuttal Fig. 3)**. Honestly, we feel that this figure is not very informative. In comparison to GTEx which had many sample types this study is small and the graph is not that helpful. We would be happy to include this figure in the Supplement if the Reviewer feels that it is helpful, but have not done so at this point.

***Comment:*** *Interpretation of some of the results is missing:
How do the authors explain the observation that "Each QTL group had numerous exact matches with eQTLs while there were few overlaps for all four QTL groups" (page 8, line 24)?*

*In general, why do the authors find so little overlap between different QTL types? It is surprising that only a very small fraction of QTLs is associated with multiple phenotypes.*

Regarding the greater overlap with eQTLs, the eQTL number is much higher than the other QTLs and this probably accounts for them having greater overlap with the other different QTLs. Regarding the general lack of overlap among the QTLs, there are a number of reasons for this, as provided below. We have now made some of these points in a modified paragraph in the Discussion section of the manuscript, which is also provided below.

1. Perhaps most important, each of the QTL datasets is almost certainly under sampling the full number of each class of QTLs, and there is stochastic sampling within each group of QTLs, thus resulting in less than valid estimation of the true overlap. In essence this may be considered as incomplete statistical power to assess the full relationship of the different QTLs.

2. caQTLs have 870 overlaps with bQTLs (Fig. 4a), and we believe this is a reasonable number. TF binding is usually associated with transcription, so it is reasonable that these exact matches have the highest fraction. And we know that TCF21 recruits factors that regulate chromatin accessibility (Zhao, Genome Med 2018).

3. The clQTLs show less match with other QTLs, and we speculate that this is because chromatin structure has a different regulatory mechanism than the more well studied effects of bQTLs, clQTLs, and eQTLs that are all related to gene transcription. This hypothesis is also consistent with our motif and histone mod data (Fig. 3).

4. QTL mapping is complicated by issues with LD, as different analyses commonly find different SNPs among groups of variants in LD. Thus exact matching of QTLs is decreased. In fact, we showed this in Fig. 4b where we identified an increase number of QTL overlaps when we extended their number by including SNPs in LD.

Page 12, line 31:

Investigation of the overlap of different classes of QTLs in HCASMC is informative. In general, the overlap of individual QTLs was modest, and likely reflects a number of features of the individual datasets. Given the limited number of subjects in these studies, it is unlikely that we were able to identify the majority of each type of QTL, which would limit overlap of the individual datasets. Also, analyses for each QTL likely showed variation in the individual SNP that might have been chosen from among a number of variants in linkage disequilibrium in an associated locus. This was demonstrated in our data, as there was significantly increased colocation of bQTLs and caQTLs when variants with linkage diseqrulibrium (R^2^>0.8) were considered (Fig. 4b), which was consistent with colocation of these QTLs across the genome (Fig. 4c). Interestingly, both of these classes of variants were also noted to be enriched at JUN binding sites, consistent with a pioneer function for the AP-1 complex (Fig. 3h) [16]. Further, enrichment of these QTLs with the smooth muscle cell MYOCD-SRF complex, SRF binding CArG boxes, indicated a role for these regulatory variants in SMC processes (Fig. 3i). Gene Ontology analysis of genes located nearby to QTLs showed high level enrichment for terms related to SMC processes such as matrix organization, cell-cell interactions - adherence junctions, and vascular development and angiogenesis. By comparison, clQTLs had less colocation and a different set of enrichment categories, suggesting an independent mechanism from bQTLs and caQTLs.

***Comment:*** *Figure 4C and its corresponding Figure S4D-F are missing statistical tests that show that "the R2 distributions of eQTL to significant (P<10-4) bQTLs, caQTLs or clQTLs was statistically higher than those to non-significant (P>0.5) QTLs" (page 8, line 44)*

We have labeled the P values in the figure. The statistical method we used was described in Methods.

***Comment:*** *Page 8, line 50: why don't the authors use Hi-C data to find potential target genes? There can be multiple ways to do this, including: finding chromatin loops with their two anchors that* *respectively overlap or are close to a TSS and a QTL; or QTLs and target genes that are included in the same TAD.*

This was a very helpful comment. We have now assigned the QTLs to their target genes using the chromosomal looping data. The gene and the QTL which are either located in the same anchor of a chromosomal loop or respectively overlapped with the two anchors of a loop were considered as a potential QTL-gene regulatory pair. Based on this approach, we identified a greater number of genes. Although the pathways identified remained the same the P-values were considerably improved. The updated analyses are shown in Fig. 4d-g and Suppl Fig.4g and the method described in the Methods section as below.

Page 19, line 39:

We used our Hi-C chromatin interaction data to assign the QTLs to their target genes. The gene and the QTL which were either located in the same anchor of a chromosomal loop or overlapped with the two anchors of a loop were considered as a potential QTL-gene regulatory pair. Gene ontology analysis was done with the PANTHER database. Pathways, biological processes, cellular component, pathways and disease enrichments were carried out using *Fisher's Exact test* and corrected by FDR 5%.

***Comment:*** *Causal loci analysis: The introductory sentence (page 9, line 15-19) seems to relate to analyses done in the previous sections. Was anything different done here?*

We have now edited the text to make more clear the direction of the studies in this section of the manuscript. The text now reads as follows:

Page 9, line 21:

To investigate the functional association of the HCASMC QTLs with expression of genes in CAD loci we pursued mechanistic studies to characterize their transcriptional relationship. Given the association of the three types of QTLs with GWAS CAD loci and the QTL target genes with SMC functions, we intersected these CAD SNPs with the QTL colocalized loci and then searched their nearest genes.

***Comment:*** *How were the 36 candidate causal genes selected (page 9, line 24)? A supplemental table describing these loci would be helpful*

The 36 causals genes were identified by their associated CAD SNPs in GWAS, CardiogramplusCAD database and related reports (Nat Genet 2017, 49:1385-1391. Circ Res 2017, 122:433-443, Nat Genet 2015, 47:1121-1130. Nat Genet 45 (2012) 25-33.). We have added an additional file 3, Suppl Table 2, which provides information on these loci.

***Comment:*** *Page 9, line 28: "most of these gene were expressed in both GTEx coronary artery tissues and HCASMC". This sentence is meaningless: are these genes more likely to be expressed in coronary artery and HCASMC than other tissues? Are they expressed at higher levels in general?*

Unfortunately, we do not fully understand this comment of the Reviewer. The point of this sentence was to simply say that we checked the expression level of these genes to be certain that they would be acceptable for the mechanistic follow-up studies.

***Comment:*** *How were the genes selected for validation (ARNTL, CCBE1, CDH13, EMP1 and FN1)? They are not the most highly expressed (several other genes have higher expression than ARNTL) and all the genes in Figure S5B-C are already known to be in CAD-associated loci.*

First, we selected these genes to verify because the HCASMC QTLs and CAD SNPs are juxtaposed to or located within the structural genes, increasing the likelihood they are functionally linked. These genes have not been validated as causal. Second, although the 3 genes (EMP1, ARNTL and CCBE1) we verified in Suppl Figs have an average expression level in GTEx tissues, the 2 most interesting genes CDH13 and FN1, which are shown in the main figure, do show high expression levels in coronary artery compared to other tissues **(Rebuttal Fig. 4).** Finally, we chose the genes with the greatest likelihood of being causal, given available eQTL data, epigenetic signatures, and chromatin accessibility, as our primary goal was to establish that disease related genes were regulated by our molecular QTLs.

***Comment:*** *CDH13 validation: the authors use CRISPRi on the clQTL (10 kb downstream of the TSS). In Figure 5A there are two anchors that overlap the variant, with their other anchors falling in introns of CDH13. What is the mechanism by which CDH13 is downregulated when the variant is silenced? The variant is 10 kb downstream of the TSS (so the anchor overlapping the variant is not at the TSS, as Hi-C has 5 kb resolution) and there are no loops connecting the variant to the TSS (if the hypothesis is that the variant overlap a regulatory element for CDH13). Also, why do the authors look for chromatin interactions between the variant and the TSS when the loop affected by the variant does not connect them? Wouldn't it be more informative to find the chromatin interactions between the anchor overlapping the variant, the other anchor (that shares the loop affected by the clQTL) and the TSS?*

Rebuttal Fig.4 The sample sizes and the QTL/target gene ratios of the TCF21 binding, chromatin accessibility, chromosomal looping, and eQTLs.

*In general, in Figure 5A, 6A and their corresponding supplemental figures, can the authors highlight which loops are affected by the clQTLs?*

The loops that are visualized in these plots that do not link to the TSS are most likely mediating enhancer-enhancer interactions. We suspect that the QTLs downstream of the TSS do indeed loop to the TSS, but we cannot visualize these loops. This is because there is a 20 kb minimal distance limit (4 times the resolution) for both *FitHiC* and *HiCCUPS* to find loops.

***Comment:*** *Discussion - Please, reformulate the sentence "The GWAS data and related insights into the mechanisms of association that are arising from such findings have spurred investigation of the relationships between genetic variation and various genomic features that relate to regulation of transcription". It is very hard to understand its meaning.*

Understood, we have modified and simplified the text to make the point clearer, the text has been edited as follows:

Page 11, line 33:

For the most part, these associations are likely mediated by variation of gene expression in these loci, due to differences in allelic variation that affects transcription in *cis*. The GWAS data and related insights into the mechanisms of association that are arising from such findings have spurred considerable interest in related mechanisms of transcriptional regulation [37]. Variants that regulate gene expression

***Comment:*** *In several places in the Discussion, the authors compare QTLs from this study to LCL QTLs. GTEx performed eQTLs on ~50 tissues, which demonstrated that many eQTLs are tissue-specific. Please, mention this in the Discussion. The analysis of the overlap between HCASMC and LCL QTLs should be moved to the Results and accompanied by figures/tables, rather than being described in the Discussion.*

As suggested by the Reviewer we have moved these comparisons to the Results section as noted below. We also now cite the GTEx publication in the Discussion section.

Page 5, line 9:

To verify the significance of our pooling and QTL mapping results, we intersected our caQTLs with the previously reported caQTLs in lymphoblastoid cell lines (LCLs) [9]. With *P* value cutoff 10^-3^, our caQTLs have 20.4% (1705) overlap with LCL QTLs. There was at most 32.9% (2745) overlap with *P* value cutoff 10^-2^, suggesting that regulatory variation is quite different between these two cell types and underscoring the importance of these smooth muscle cell data for study of vascular disease genetics.

***Comment:*** *Page 12, line 28: "The finding of colocalization of different classes of QTLs in HCASMC is highly informative". The overlap between different QTL types is very low: how do the authors justify this as highly informative?*

As discussed above, there are many reasons why the overlap does not represent the dominant portion of each group of QTLs. We would respectfully argue that the overlaps that we do see are informative. The fact that there is minimal overlap of the clQTLs with other QTLs is interesting, and must reflect a fundamental difference in their functional basis. Also, the bQTL and caQTL colocalizations were directional and established that TF binding can promote open chromatin. We based our functional analysis on the colocalizing QTLs, and our validation of such multi-functional QTLs has not been done previously in such a comprehensive fashion.

***Comment:*** I could not find a statistical analysis supporting the statement "In our data there was significant colocalization of bQTLs with caQTLs, and both of these with eQTLs" (page 12, line 35).

This sentence was deleted and replaced with more specific and circumspect comments regarding the QTL overlaps, as noted above.

***Comment:*** *Page 13, line 26: "For those QTLs that overlap, especially for overlapping bQTLs and caQTLs, there is an increased likelihood that these variants are the active regulatory variant in the region, and thus more likely to contribute to the risk association in the associated locus". How was this measured?*

Since we do not actually know the causal variant in these loci, we have not done formal CRISPR or other studies to investigate this, we cannot know what is the functional variant in the region, so we used this approach to increase the likelihood that the variants we chose are indeed causal. So, in short, there is not a way to evaluate the effectiveness of this approach since we do not know the causal variant.

***Comment:*** *Methods: Mapping and analyzing QTLs: how was the sample source (samples were purchased from 3 different sources) taken into account for this analysis? What about other covariates (ethnicity and sex for example)?*

We added an additional Supplemental Table 3 (Additional file 4) with samples source, pooling and subject information. We do not think that these variables would change the QTL analyses presented here, as they might be expected to affect eQTLs. Also, the numbers are too small to expect that we could find relationships to these covariates.

***Comment:*** *Page 17, line 52: "In this way, pre-allele frequencies already account for some types of trans-acting variation, increasing the power for mapping cis-acting differences". Please, explain what this sentence means and what is intended by "trans-acting variation".*

We have added text as follows to better explain this sentence, and this text now appears in the Methods section.

Page 18, line 17:

In this way, pre-allele frequencies already account for some types of trans-acting variation, increasing the power for mapping *cis*-acting differences. For example, if one cell line has a greater abundance of TCF21, leading to widespread increased TCF21 binding, then its alleles will be over-represented in the post-ChIP reads. By estimating the pre-ChIP allele frequencies directly from the post-ChIP reads, this variation in TCF21 abundance is accounted for, since it will affect the pre- and post-ChIP allele frequencies equally [13].

***Comment:*** *GWAS overlap: without knowing the ethnicity of the individuals, it is impossible to know if LD should be calculated based on the CEU population.*

As we described in Suppl. Table 3, there are 52 Caucasian, 11 African Americans, 6 Hispanic, 2 Asian, and 1 unknown. We feel that using the CEU population is the best approach we can use to calculate LD.

***Comment:*** *Motif analysis: "These results were further validated by MEME". How was this done and where in the results is this described?*

The MEME analysis details have been added to the Methods section.

***Comment:*** *Statistical analysis: FDR corrections are missing throughout the text.*

For qPCR tests, peak and loops calling, we used FDR 1%, for HOMMER motif analyses, we used FDR 0.1%, for all other multiple comparison testing and gene ontology enrichment analyses, we used the default FDR 5%. We have updated this information in the Methods section.

For FDR in the regression study, it is hard to randomize the allele frequency tests, therefore we didn’t use an FDR cutoff to adjust P values in the regression analysis. When using current 1e-3 p value cutoff, the actual FDRs are 9.01% for bQTL, 1.89% for caQTL and 0.35% for clQTL.

**Second round of review**

**Reviewer 2**

The authors did a great job addressing comments/concerns.  The manuscript is much improved.
